# On-the-Fly Microfluidic Control of Giant Vesicle Compositions Validated by DNA Surface Charge Sensors

**DOI:** 10.1021/acsnano.4c16289

**Published:** 2025-04-04

**Authors:** Marcus Fletcher, Yuval Elani

**Affiliations:** Department of Chemical Engineering, Imperial College London, Exhibition Road, London SW7 2AZ, United Kingdom

**Keywords:** microfluidics, membrane surface charge, lipid composition, DNA nanotechnology, Giant Unilamellar Vesicles

## Abstract

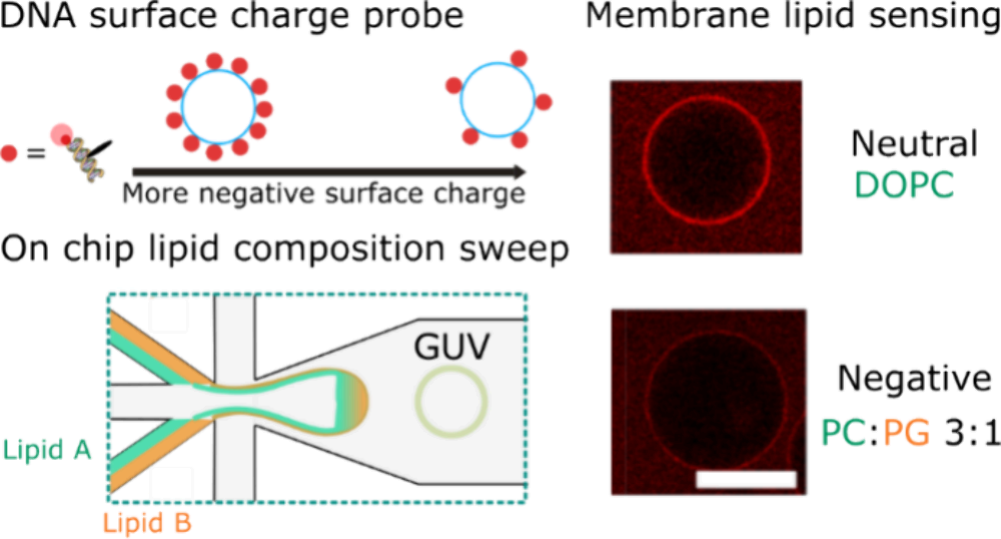

The specific lipid composition of cell membrane microenvironments plays a critical role in regulating a range of cellular processes such as integral and peripheral membrane protein function, cell morphology, and membrane potential. However, harnessing similar complex capabilities in artificial membrane mimics remains challenging. In large part, progress has been slow due to a scarcity of techniques for both (i) accurately quantifying composition-dependent properties of artificial cell models at the single-vesicle level and (ii) efficiently exploring large multidimensional composition spaces. Here, we address both challenges by first developing an assay for quantitatively sensing giant unilamellar vesicle (GUV) membrane surface potentials using a fluorescent cholesterol-labeled DNA duplex sensor. We then devised a microfluidic vesicle assembly line enabling the continuous, on-chip production of lipid vesicles with variable compositions. This enabled real-time, on-the-fly adjustment of membrane compositions and biophysical properties as vesicles were being produced, followed by membrane analysis using our assay. Analysis of the association of our DNA fluorescent probe with single vesicles reveals that we may quantify the surface potential of vesicle membranes *in situ* through quantification of the membrane-probe binding constant. Our work paves the way for the production and biophysical analysis of artificial cell libraries that can enable rational artificial cell engineering.

Reverse engineering the functions of living cells by constructing synthetic cell mimics has long been a goal of bioengineering. In the last decades, many cell functions including protein expression,^1−4^ energy generation,^5^ sensing of external stimuli,^6^ and cellular division^7^ have been demonstrated in cell models formed from molecular building blocks, termed artificial cells (or synthetic cells/protocells). Artificial cells can be used as simplified models to understand biology away from the complex milieu of living systems. They are also increasingly being recognized for their potential in biotechnology, as programmable bioderived micromachines for application in healthcare, sensing, energy, and beyond.^8^

Typically, artificial cells are based on cell-sized, lipid-membrane-bound aqueous compartments called giant unilamellar vesicles (GUVs). In recent years, the cytosolic processes in artificial cells have been rapidly increasing in complexity through inclusion of protein expression systems, genetic circuits,^6^ internal transport modules,^9^ and embedded subcompartments.^1,10^ Mimicking cell membrane functions, on the other hand, remains challenging despite the enormous potential of recapitulating processes at the membrane.

Membranes serve vital roles in an array of complex functions and behaviors essential to life, including signaling, division, endo- and exocytosis, and multicellular communication. However, endowing artificial cells with similar capabilities has been difficult due to our current lack of insight into how to translate mechanistic pictures into design rules for synthetic biological membranes. Accordingly, our ability to unravel membrane composition-function relationships needs to be addressed for the ambitions of bottom-up synthetic biology to be realized.

There are two bottlenecks limiting exploitation of membrane engineering design principles: (i) membrane compositional spaces are vast, yet we still do not possess methods to quickly vary the membrane composition of artificial cell models as they are being continually produced. (ii) While mechanistic insight may be facilitated by quantitative comparison of theoretical models of membrane processes, there are only a handful of techniques for accurately quantifying membrane biophysical parameters of artificial cells. Here, we address both of these bottlenecks by first developing an assay to quantify a key bioelectrical property of artificial cells: membrane surface charge density. Consequently, we use our new assay to characterize a microfluidic method for “on-the-fly” variation of GUV lipid composition, where lipid composition can be changed in real time, during the on-chip vesicle assembly process, simply by changing the operating parameters of the device itself.

We chose to probe membrane electrical properties as, unlike permeabilities and mechanical responses, for example, techniques for the quantification of parameters like surface charge density of single artificial cells are particularly lacking. Indeed, to our knowledge, there are no widely accepted techniques for accurately quantifying surface charge density metrics, like surface potentials, at the dimension scale of giant vesicles (diameters of c. tens of micrometers).^11−13^ Yet, in native cells, electrical properties of the lipid membrane such as surface charge density are implicated in many complex cellular processes. For example, lateral surface charge distributions are correlated: with mammalian cell membrane morphology dynamics,^14^ regulation of ion transporters and GPCRs,^15,16^ and protein association.^17^ Therefore, recapitulating and studying such membrane electrical phenomena in artificial cell systems may reveal the biophysical mechanisms underlying lipid/protein electrical interactions and, consequently, uncover critical as yet unexplored engineering principles for increasing artificial cell membrane complexity of function. However, in the first place, new probes to quantify membrane electrical properties such as surface charge density are needed.

Here, we employ a DNA duplex-based membrane surface charge density sensor (1C-DNA), modified with cholesterol (1C) and fluorescent motifs (Cy5) to promote and quantify membrane association, respectively.^18^ We demonstrate that 1C-DNA membrane association obeys a simple Langmuir isotherm for vesicles of varying anionic surface charge and that the associated binding constant, *K*_B_, is dependent on membrane surface charge density. We then demonstrate that through measurements of *K*_B_, we may extract membrane surface potential estimates proportional to the zeta potential, a widely used metric of surface charge. While other attempts have been made to quantify useful surface potentials like zeta potential on cell-sized artificial cell models with dynamic light scattering (DLS), accurate quantification suffers from the fundamental limitation that micron-scale vesicles have extremely low diffusion constants and, consequently, difficult to interpret correlation functions.^11,12^ Moreover, DLS cannot be used to derive single artificial cell measurements to probe distributions of the membrane charge. DLS limitations may be partially addressed by the use of cell-penetrating peptide binding experiments to study membrane surface charge; however, membrane active peptides can compromise membrane integrity.^13,19,20^ In addition, to enable the high-throughput generation of vesicles with varying lipid compositions, we design a microfluidic vesicle assembly line to vary the lipid content of vesicles being produced on-the-fly. To validate on-the-fly microfluidic lipid composition control, we employ our surface charge probe to demonstrate mixing lipid phases on-chip produces vesicles with the same compositions as lipid solution inputs prepared one by one using traditional approaches. Establishing the principle of microfluidic preparation of multilipid compositions paves the way for the generation of artificial cell membrane composition libraries.

## Results and discussion

### Sensing Vesicle Membrane Surface Charge Density via DNA Probe Binding Measurements

In designing a technique for sensing membrane charge, we considered the following guiding principles: (1) single-vesicle resolution, (2) high scalability to parallel measurement, and (3) low technical requirements. We reasoned that a fluorescence-based method could fulfill these three requirements as large samples of single vesicles may be measured in parallel, under standard microscopy equipment for biological laboratories. DNA nanotechnology constitutes a richly flexible platform for designing fluorescent-based probes for a range of sensing applications.^21^ Moreover, DNA nanostructures can be engineered to associate to lipid membranes by inclusions of hydrophobic anchors such as cholesterol or porphyrin.^18,25−27^ In addition, due to its strong electrostatic charge, we anticipated that DNA bound to membranes via hydrophobic anchors may be useful for sensing membrane surface charge density. Indeed, Ochmann et al. developed a single-molecule Förster resonance energy transfer (FRET) sensor comprising a large DNA origami plate anchored to lipid vesicles via an array of cholesterol modifications, with which they could sense variations in anionic lipid fraction via a change in FRET signal.^28^ Nevertheless, the high technical requirements of single-molecule FRET setups as well as construction and purification of a DNA origami limit the usefulness of this sensor for most artificial cell research laboratories. Instead, we investigated whether similar charge density sensing measurements could be achieved for cell-sized vesicles with a simple DNA duplex-based sensor.

**Figure 1:**
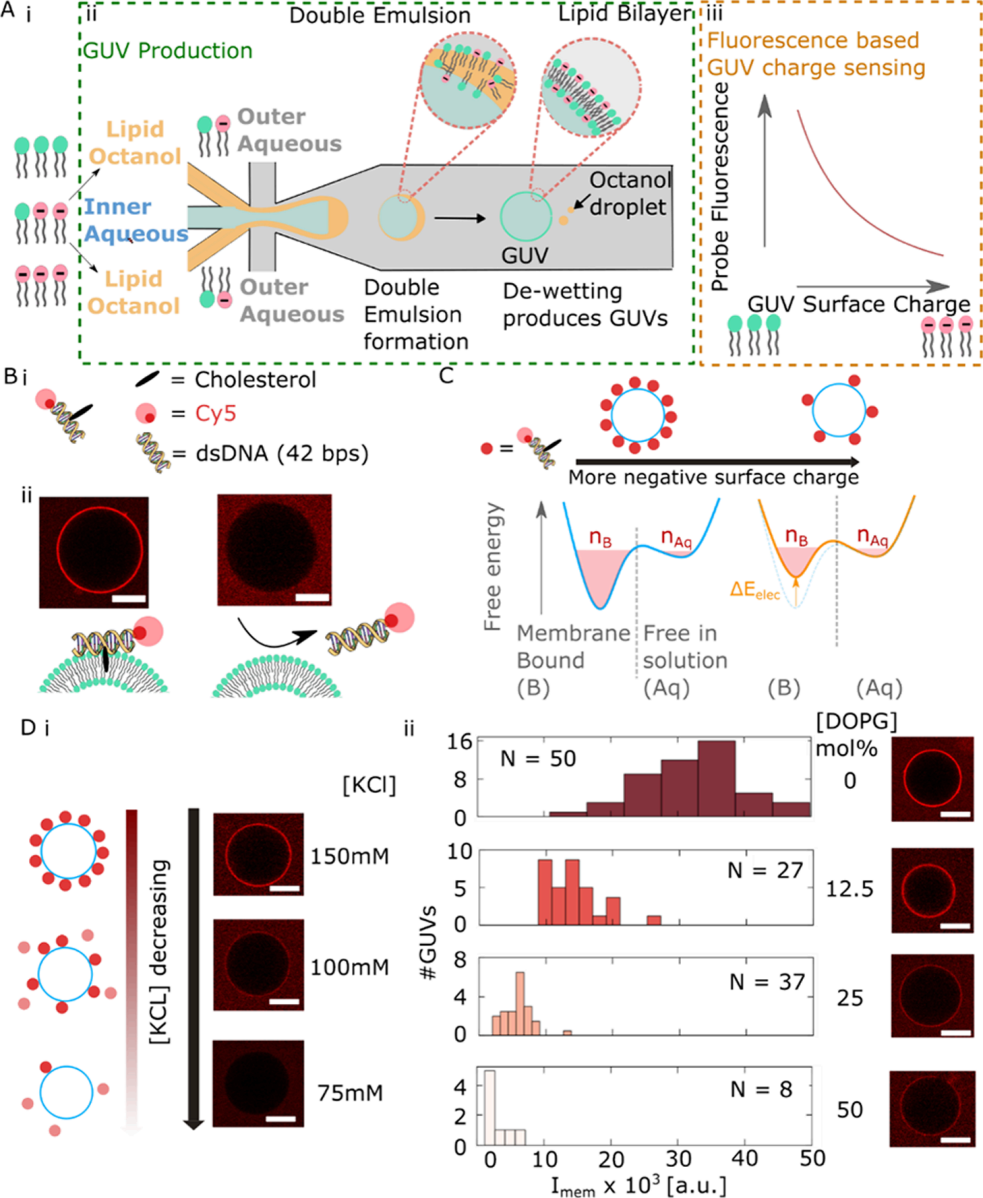
Principle of membrane surface charge sensing using fluorescently modified DNA nanostructures. (A) (i) First, we prepare lipid stock phases (LO) with different proportions of anionic lipid (DOPG). (ii) For each lipid-octanol stock, we perform microfluidic production of vesicles using octanol-assisted liposome assembly.^21−24^ Vesicles are formed after dewetting of W/O/W (inner aqueous (IA)/lipid octanol (LO)/outer aqueous (OA)) double emulsion droplets. (iii) To complete our surface charge density quantification pipeline, we aimed to design a fluorescent readout for the anionic lipid content by creating a fluorescent membrane associating surface charge density probe. (B) (i) Schematic of the fluorescent membrane associating the DNA probe. A 48-base pair DNA duplex is modified with a fluorophore (Cy5) at one end. In order to promote membrane association, a cholesterol modification is added 12 base pairs along the duplex. (ii) Confocal micrographs demonstrating membrane association due to cholesterol modification. (C) Illustration of the effect of electrostatic repulsion due to membrane surface charge density on DNA sensor binding. (D) (i) Decreasing the electrostatic salt screening by reducing the ionic strength strongly reduces binding of the DNA probe ([DNAadded] = 500 nM) to the membrane (DOPC). (ii) Equivalently, maintaining the electrostatic screening but increasing the membrane surface charge density by including greater amounts of anionic lipid (DOPG) reduces the binding of the DNA probe ([DNAadded] = 500 nM) to the membrane in 500 mM glucose PBS. Scale bars = 10 μm.

To design our sensor, we chose a short (48 base pair) double-stranded DNA modified with a fluorophore (Cy5 or Atto532 or Atto647) at one end, and a cholesterol anchor joined via a TEG linker 12 base pairs along (1C) from the other end (Figure 1B(i)).^18^ To validate the probe design, we formed vesicles (DOPC) from microfluidics using octanol-assisted liposome assembly (OLA) (illustrated schematically in Figure 1A(i)) (encapsulating 500 mM sucrose PBS pH 7.4) and incubated them with the DNA probe (500 nM 1C-DNA-Cy5, 500 mM glucose PBS pH 7.4). Using a sucrose/glucose asymmetry across the membrane, we settled vesicles down under gravity to the bottom of an incubation well and observed the fluorescent accumulation at the membrane due to association of the DNA probe (Figure 1B(ii)). We found a strong association of 1C-DNA-Cy5 fluorescence to the membrane with a single cholesterol modification (Figure 1B(ii) (left)) but little or none without cholesterol (Figure 1B(ii) right), as reported by previous studies,^18^ suggesting that fluorescent labeling does not significantly affect DNA binding to lipid membranes.

Next, to investigate whether 1C-DNA-Cy5 membrane binding interaction was sensitive to membrane electrostatic repulsion, we repeated the incubation of DOPC vesicles with our probe with solutions of different ionic strength down to 75 mM KCl (Figure 1D(i)). As the salt concentration decreases, thereby decreasing the screening of electrical charge by ions, we found a significant decrease in membrane-associated 1C-DNA-Cy5 (Figure 1D(i)). Importantly, similar DNA duplex probes were found to be stably assembled down to 10 mM ionic strength, so we can discount disassembly of the probe as a cause of decreased binding. Consequently, our observations may be rationalized by considering the interaction between 1C-DNA-Cy5 and the lipid bilayer as a reversible two-state process with an associated equilibrium binding constant, as illustrated in Figure 1C.

The free energy change of binding includes an electrostatic component: ε_elec_. By decreasing the salt screening, ε_elec_ between the strongly negatively charged DNA duplex and the weakly negatively charged vesicle membrane (DOPC, zeta potential ∼ −4–17 mV) increases.^29,30^ In turn, higher ε_elec_ increases the free energy of the membrane-bound state while leaving the unbound state unaffected, resulting in a less negative Δ*G*, for the membrane binding process. Consequently, fewer 1C-DNA-Cy5 molecules are bound at equilibrium, encapsulated by a smaller *K*_B_. This reasoning is illustrated schematically in Figure 1C.

In principle, ε_elec_ ought also to depend on the membrane surface charge density for a given salt concentration. Therefore, measurements of ε_elec_ may allow quantification of the membrane surface charge density. We tested whether we could sense variations in the membrane surface charge density at physiological salt concentrations (PBS) using DNA probe binding measurements. Using our microfluidic generation method, we prepared vesicles with varying proportions of zwitterionic DOPC and anionic DOPG lipid from 0 to 50 mol % DOPG (see Experimental Methods) and measured 1C-DNA-Cy5 probe binding (Figure 1D) for populations of individual vesicles. The histograms of vesicle membrane fluorescence display a decreasing trend as an increasing fraction of DOPG is included in the membrane, indicating that 1C-DNA-Cy5 binding measurements may be used to sense the membrane surface charge density.

### 1C-DNA-Cy5 Probe Binding Fits a Two-State Binding Model

In the last section, we demonstrate that 1C-DNA-Cy5 binding measurements may be used to sense the vesicle anionic lipid fraction. To extract a metric for quantitative comparison of membrane surface charge density between vesicles, it is necessary to first model the binding interaction between the vesicle membrane and DNA probe.

**Figure 2:**
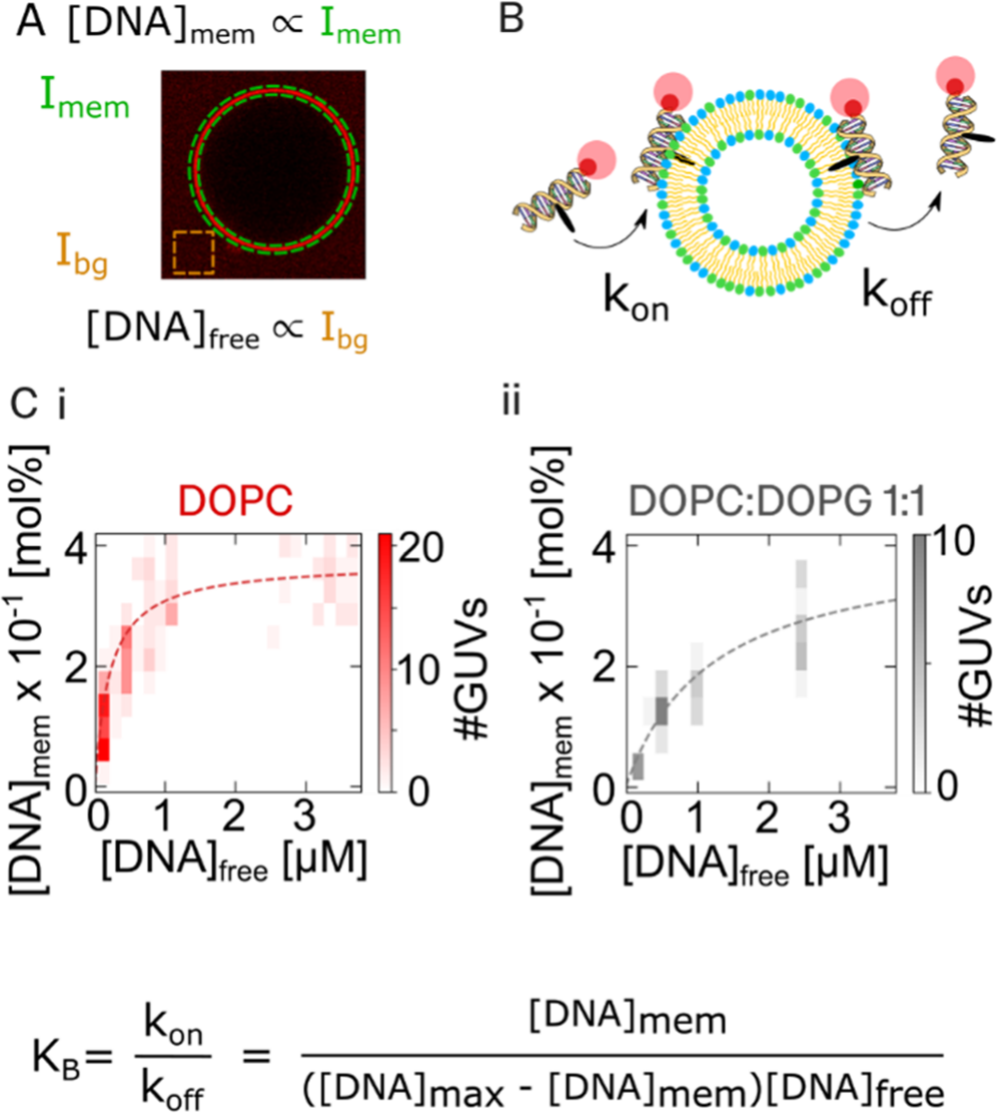
1C-DNA probe binding follows a Langmuir isotherm model. (A) Fluorescent quantification of membrane-bound and free DNA concentrations. Schematic depicting fluorescence-based quantification of membrane-bound DNA and free DNA concentrations using confocal microscopy. Measured Cy5 fluorescence at the membrane (*I*_mem_) and in the aqueous environment (*I*_bg_) were converted to DNA concentration [DNA]_mem_ and [DNA]_free_ using a calibration of fluorescence vs Cy5-PE and Cy5-DNA concentration, respectively (Figure S3). (B) Schematic of the 1C-DNA membrane binding process as a simple two-state model, with associated transition rate constants *k*_on_and *k*_off_. (C) (i) 2D histogram of membrane-associated 1C-DNA [DNA]_mem_ and free 1C-DNA concentrations [DNA]_free_ for single DOPC GUVs. A Langmuir isotherm model (eqs 1–3) was fit to these distributions (red dotted). (C) (ii) Similar 2D histogram of [DNA]_mem_ and [DNA]_free_ for single DOPC:DOPG 1:1 molar ratio GUVs with associated Langmuir isotherm model fit (gray dotted). From these fits, we derived estimates for the apparent binding constant, *K*_B_, and maximum DNA concentration [DNA]_max_ for each lipid composition, revealing a significant dependence of DOPG mole fraction on *K*_B_ but not on [DNA]_max_.

To confirm the validity of this model, we measured a binding curve by incubating vesicles in custom-made imaging chambers (see Experimental Methods) with different concentrations of 1C-DNA-Cy5, [DNA]_added_. For each sample, the binding process was allowed to reach equilibrium by incubating for 2–3 h before imaging. The mean intensity of pixels on the membrane, *I*_mem_, as well as the background intensity locally to each vesicle, *I*_bg_, were measured (see Experimental Methods), as illustrated in Figure 2A. We converted background-subtracted *I*_mem_ and *I*_bg_ into membrane-bound DNA molar percentages, [DNA]_mem_, and free DNA concentrations, [DNA]_free_, by calibration (shown in Figure S3 and method described in Supporting Information S3). Note that [DNA]_mem_ is measured as a molar ratio of DNA:lipid. Figure 2B displays histograms of [DNA]_mem_ for populations of vesicles with the concentration of [DNA]_added_ displayed, showing the increase of [DNA]_mem_ in accordance with le Chatelier’s principle.

To evaluate the suitability of a Langmuir isotherm model to 1C-DNA-Cy5 binding to vesicles, we fit the following equation to the mean [DNA]_mem_from the vesicle distributions and the measured [DNA]_free_ values in each case (Figure 2C):
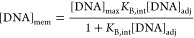
1^31,32^where [DNA]_max_ is the maximum [DNA]:[lipid] bound to the membrane, *K*_B,int_ is the intrinsic binding constant between 1C-DNA and the lipid bilayer, and all other parameters are as previously defined. [DNA]_adj_ is related to the measured free DNA concentration, [DNA]_free_, by the following equation:
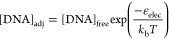
2where *k*_b_ the Boltzmann constant, *T* is the temperature, and ε_elec_ is the electrostatic energy due to repulsion between 1C-DNA and the membrane surface charge at its position adjacent to the bilayer. Similar to established models considering binding of charged species to lipid bilayers, eq 2 accounts for the Boltzmann distribution of DNA from the charged lipid bilayer due to electrostatic interactions.^33,34^ As Δε_elec_ ought to be consistent for a given lipid composition, we may define an effective Langmuir binding constant, *K*_B_, related by
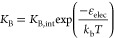
3

Consequently, *K*_B_ and [DNA]_max_ were both used as fitting parameters. For DOPC vesicles (red, Figure 2C), we found an excellent fit of the model (eq 1), implying that the binding process may be adequately described by a two-state binding process characterized by a single *K*_B_. Notably, studies of other binding of similar cholesterol-modified DNA nanostructures and zwitterionic lipid membranes have reported Langmuir-like binding.^31^ The distributions in both Figure 2C(i) and Figure 2C(ii) are broad, however, implying a wide variation of DNA binding between individual GUVs. This may indicate varying concentrations of residual octanol in the membrane from the preparation, as octanol could influence the surface charge density of the membrane. However, wide distributions in GUV population data have been found in single-particle measurements of GUVs made from octanol-free methods.^21,22^ It is also possible that the variance in single GUV [DNA]_mem_ measurements may be explained by higher-order models that account for DNA–DNA interactions and mechanical couplings.

We estimated for DOPC vesicles that *K*_B_ = 4.7 ± 0.8 μM^–1^, and [DNA]_max_ was 0.37 ± 0.02 mol % (where mol % ). Interestingly, our measured [DNA]_max_ is similar to the value obtained when considering maximal packing of freely rotating 1C-DNA probes (see the Supporting Information), indicating that the 1C-DNA binding may be limited by the number of membrane binding sites rather than interactions between bound DNA molecules.

Next, we repeated the DNA binding curve titration with negatively charged vesicle membranes prepared with a lipid composition of 1:1 DOPC:DOPG (gray, Figure 2C). As with the DOPC vesicles, we found that a Langmuir isotherm binding model gave a good fit to the experimental data. From the fitting process, we derived values of *K*_B_ = 0.82 ± 0.21 μM^–1^ and [DNA]_max_ = 0.4 ± 0.05 mol %. Interestingly, for both DOPC and DOPC:DOPG 1:1 membranes, the [DNA]_max_ value extracted agrees within the error, suggesting that in both cases, the maximum DNA attachment may be limited by available space on the membrane. Importantly, as the effect of increasing membrane surface charge density appears to affect *K*_B_ only (an order of magnitude decrease with 50 mol % DOPG inclusion) and not [DNA]_max_, we propose that measurements of *K*_B_ alone are sufficient to quantify the membrane surface charge density.

### Quantitative Measurements of 1C-DNA Binding to Vesicles of Different Membrane Surface Charge Density

**Figure 3:**
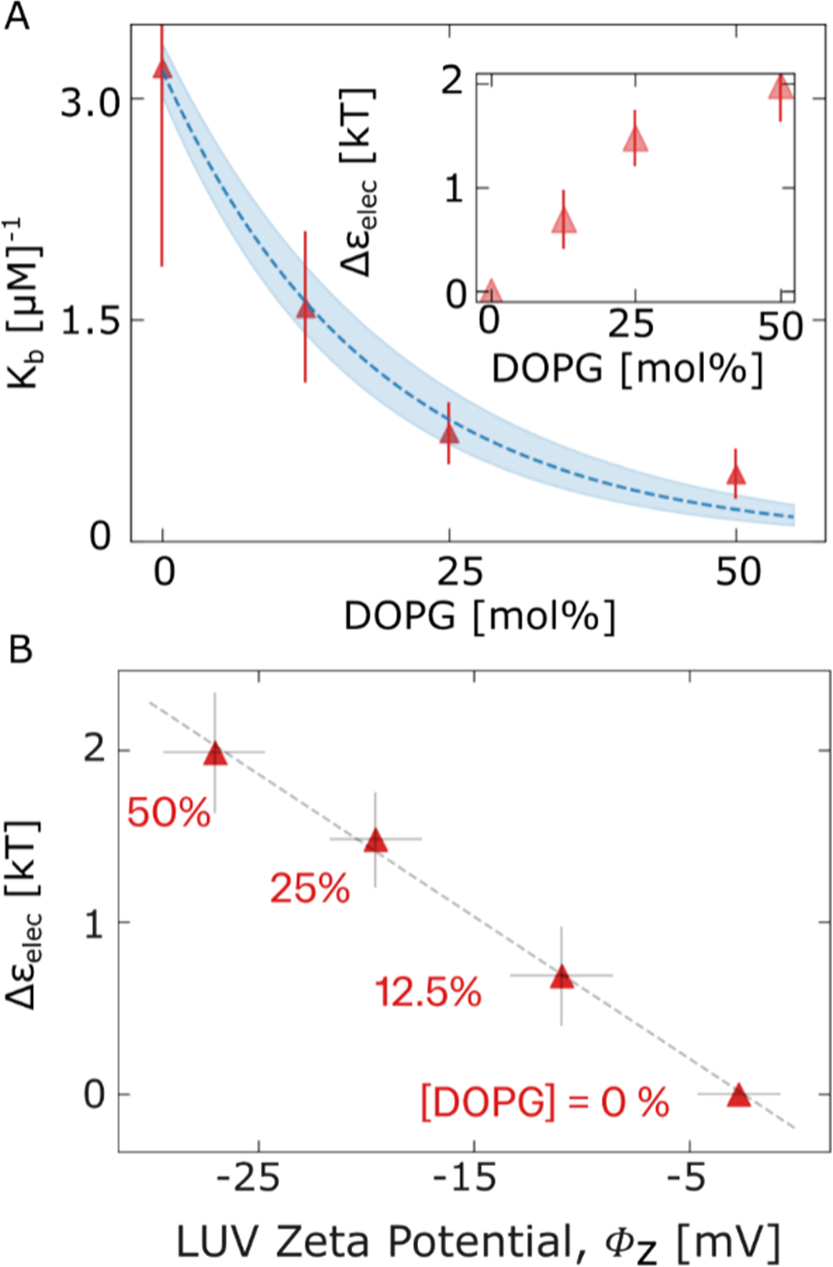
1C-DNA probe binding measures membrane surface potential. (A) Plot of mean 1C-DNA/vesicle binding constant, *K*_B_, for vesicles prepared with different molar fractions of anionic DOPG in the OLA lipid-octanol phase (see Experimental Methods). An exponential fit is shown (blue), implying a linear relationship between free energy change and DOPG content. From the fractional change in *K*_B_ due to increasing DOPG fraction, we can derive the change in associated electrostatic repulsion energy, Δε_elec_ (see Section S7) shown inset. (B) Plot of Δε_elec_ derived from GUV/1C-DNA binding constants against zeta potential, ϕ_z_, of LUVs prepared with the same DOPG mole fraction (see Experimental Methods). A linear relationship between these quantities (dotted line), which each have nonlinear dependencies on DOPG mole fraction, implies similar surface charges for OLA GUVs and LUVs prepared with the same DOPG content.

To elucidate the relationship between *K*_B_ and the membrane surface charge density, we used our binding model from the previous section to extract estimates of *K*_B_ for vesicles of each DOPG fraction prepared (see Figure 1). As before, we resolved [DNA]_mem_ and [DNA]_free_ from *I*_mem_ and *I*_bg_, respectively. Then, *K*_B_ values for each vesicle were estimated by using the following expression:
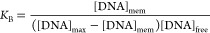
4where [DNA]_max_ = 0.4 mol % and independent of DOPG content as discussed in the previous section. Figure 3A displays the relationship between the mean *K*_B_ over vesicle populations and the DOPG molar fraction, *n*_PG_. *K*_B_, displayed in Figure 3A, appears to decrease monoexponentially with increased DOPG fraction *n*_PG_, leading us to define the empirical relationship:
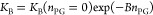
5where *B* = 3.2 ± 0.2. Given our hypothesis that an increase in *n*_PG_ leads to an increase in membrane surface charge density, we identify the physical cause of the decrease in *K*_B_ as the increase in electrostatic repulsion energy, Δε_elec_, that causes a depletion of 1C-DNA adjacent to the membrane (see Figure S7).

We may relate *K*_B_ to Δε_elec_ using the following relation:
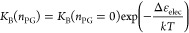
6where *k* is the Boltzmann constant and *T* is 298 K and extract estimates for Δε_elec_ (see the Supporting Information (SI) for details of derivation). Figure 3A (inset) shows an approximately linear dependence of Δε_elec_ on the membrane DOPG fraction, *n*_PG_, at low fractions (*n*_PG_ < 25%), with a significant deviation after. This result may be explained by considering the theoretical prediction of the widely adopted Grahame equation, in which the surface charge (proportional to *n*_PG_) is expected to be proportional to the surface potential, ϕ_s_, for low potentials (ϕ_s_ < 25 mV), but for large potentials, ϕ_s_ deviates beneath this trend.^35^

### 1C-DNA Membrane Binding Repulsion Estimates Are Proportional to Zeta Potential

Next, we compared our derived values for Δε_elec_ from 1C-DNA binding to GUVs with zeta potential measurements of LUVs with equivalent DOPG mole fractions, revealing an approximately linear relationship between these two quantities (see Figure 3B). Given the nonlinear dependence of both these quantities on lipid composition, their linear proportionality suggests that the OLA GUVs retain the same lipid composition as those prepared in the LO phase.

By modeling the electrostatic interaction between the vesicle membrane and the 1C-DNA probe, we derived a relationship between Δε_elec_ and the increase in surface potential, Δϕ_s_, associated with increasing *n*_PG_:
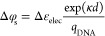
7where *k* is the Boltzmann constant, *T* is the temperature (298 K), *q*_DNA_ is the total charge of the 48 base pair duplex = 24e,^36^ κ is the inverse Debye length, λ = 0.78 nm,^28^ and *d* is the separation between the duplex center of mass and the surface that has potential, ϕ_s_.Equation 7 rationalizes the linear relationship between our measured Δε_elec_ values and ϕ*_*z*_* and implies that we may estimate the zeta potential of the membrane, if we know the separation of the DNA center of mass from the membrane slipping plane. Alternatively, we can estimate the separation of the 1C-DNA probe from the slipping plane by fitting eq 7 to the trend in Figure 3B. From the fit in Figure 3B, we derived estimates for the distance between the bound 1C-DNA probe and the slipping plane, *d* = 1.93 ± 0.03 nm. Our value for *d* is similar to predicted equilibrium separations of 1C-DNA from the membrane stern layer derived from simulations of similar probes (*d*_pred_ ∼2 ± 0.3 nm,^31^ suggesting that our measurements indeed quantify a surface potential close to the membrane. Notably, our estimate for the 1C-DNA probe separation is smaller than the predicted separation from the stern layer, which is a plausible position for the slipping plane, meaning that using our estimate for *d* may make eq 7 a feasible relationship with which to derive the GUV zeta potential. Future studies, which measure the separation from the membrane surface, will allow more precise measurements of defined membrane surface potentials.

### Quantitative Membrane Anionic Lipid Fraction Validates on-the-Fly Microfluidic Lipid Composition Control

**Figure 4:**
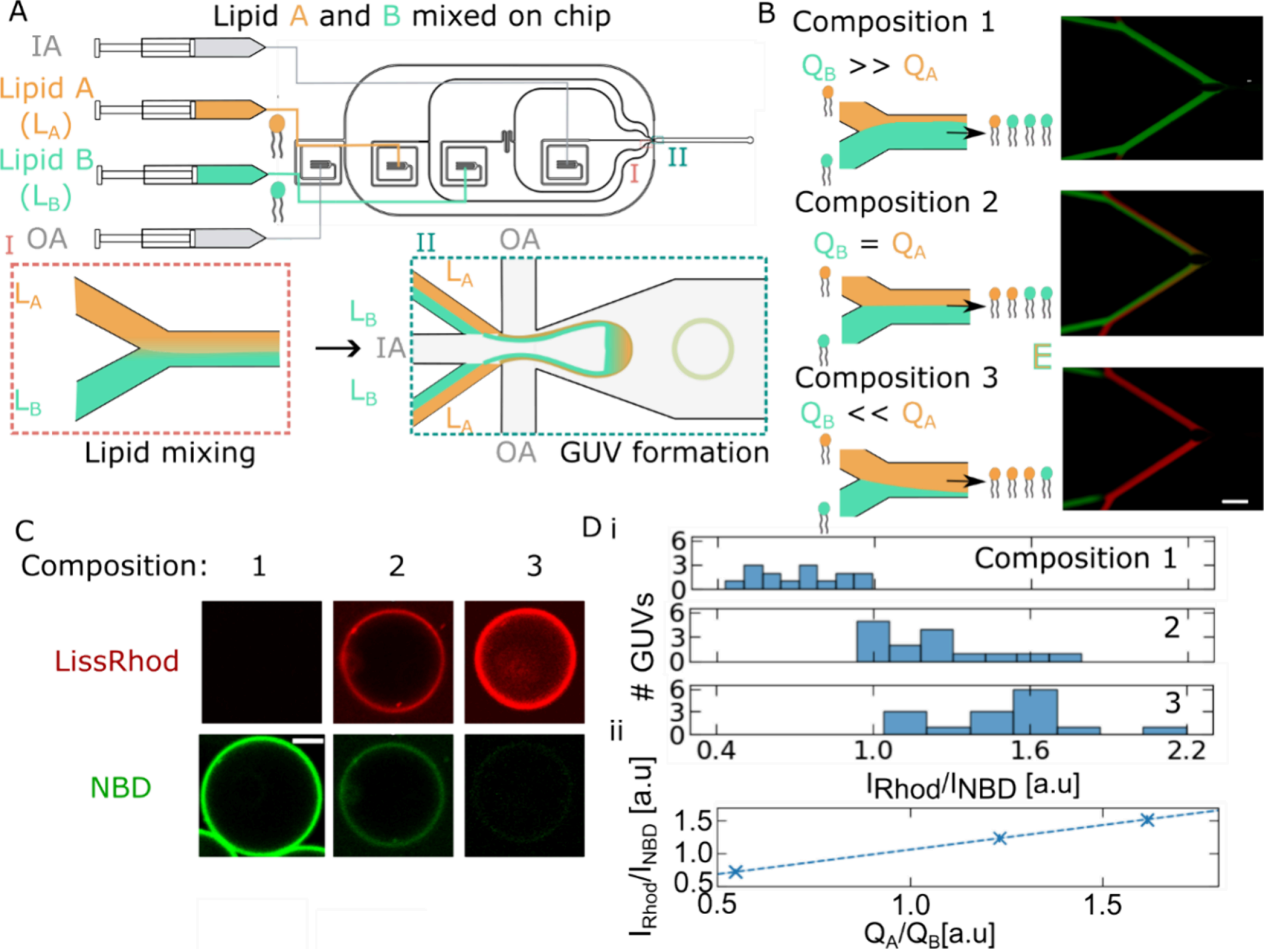
Development of microfluidic “on-the-fly” lipid composition control of vesicles. (A) Illustration of the microfluidic setup for “on-the-fly” lipid composition control. We modified the octanol-assisted liposome assembly (OLA) protocol to include one additional lipid-octanol phase, leading to four fluidic inlets: inner aqueous (IA), lipid A in 1-octanol (LipidA), lipid B in 1-octanol (LipidB), and the outer aqueous phase OA. (I) We include microfluidic mixing junctions immediately prior to the vesicle formation junction (II) such that changing the relative flow rates between LipidA and LipidB results in different lipid-octanol phase flows at the point of vesicle formation. (B) Using fluorescently tagged lipids (LipidA): Liss Rhod-PE (red) and (LipidB): NBD-PE (green), we demonstrate that lipid-octanol phases reaching the vesicle formation junction can be modulated by changing the flow rates, *Q*_*a*_ and *Q*_*b*_. A video of this demonstration is included as Supplementary Video 1. We display here three different flow rate conditions leading to three distinct compositions 1, 2, and 3. Scale bar: 100 μm. (C) For vesicle samples produced with lipid-octanol compositions 1, 2, and 3, we imaged vesicle samples using confocal microscopy. Representative images are shown. For each vesicle, we measured the membrane intensity in the Liss Rhod (*I*_Rhod_) and NBD (*I*_nbd_) channels, (see Experimental Methods for the optical setup). Scale bar: 10 μm. (D) (i) Distributions of relative intensity in rhodamine (*I*_Rhod_) and NBD (*I*_nbd_) channels of single vesicles produced at varying LipidA to LipidB flow rates, *Q*_*a*_ and *Q*_b_, respectively. Linear relationship between the mean ratio of *I*_Rhod_:*I*_nbd_ membrane fluorescence and the relative flow rate of lipid composition during production (see Section S7 for determination of relative flow rates).

In addition to the lack of in situ probes of vesicle membrane composition, understanding membrane composition function relationships and engineering artificial membrane complexity is equally hampered by labor-intensive vesicle production techniques. Typical benchtop techniques for producing vesicles such as the inverted emulsion technique or electroformation require several hours to produce vesicles.^37,38^ Thus far, varying the lipid composition of vesicles produced by these techniques necessitates human-led repetition of the production process, which is time costly. Conversely, producing vesicles with a range of lipid compositions could be achieved rapidly on a microfluidic device by employing Y junctions that mix together multiple lipid stock phases (see Figure 4A(I)). By varying the relative flow rates of several lipid-octanol (LO) phases, one can tune the final lipid composition exiting the mixing junction, which then serves as the lipid-octanol phase composition for the vesicle formation junction, as illustrated in Figure 4A(I and II). In principle, lipid compositions could be defined by the relative flow rates of computer-controlled pumps for each stock solution, enabling libraries of vesicle lipid compositions to be formed without human intervention. Equivalent approaches for mixing water-in-oil droplet compositions have now inspired a revolution in high-throughput parameter screens of aqueous biological systems.^39^

However, to our knowledge, the mixing of lipids in oil phases, which may form micellar structures, on-chip has not been explored and so is poorly understood. Moreover, while the bulk preparation (>10s μL) of lipid stocks may be well mixed advectively before lipid film formation, mixing of lipids on a microfluidic device must be limited by diffusion due to the laminar flow condition. As such, differences in diffusion times between lipids may result in unexpected final lipid-octanol phase compositions at the vesicle formation junction. Therefore, to explore the feasibility of controlling the lipid composition of vesicles on chip, we used our probe of membrane anionic lipid composition to compare the composition of vesicles formed from lipid-octanol phases prepared “off-chip” to those prepared using “on-the-fly” lipid composition control.

### Development of a Microfluidic Device for “On-the-Fly” Lipid Composition Control

To control the composition of lipid-octanol phases on a chip, we developed a new microfluidic device based on the octanol-assisted liposome assembly method, which we display in Figure 4i.

By adding a lipid mixing junction upstream of the vesicle generation module, and adjusting the flow rates of different lipid-containing inlets at this junction, we were able to produce vesicles with varied compositions. The original method is described in detail in the Experimental Methods section. Briefly, it requires the mixing together on a chip of three fluidic phases: inner aqueous (IA), lipid-octanol (LO), and outer aqueous (OA) phases were used in a fluid regime to produce vesicles. We modified the device design to include an additional lipid-octanol phases such that two lipid-octanol phases, LipidA (orange) and LipidB (green), containing lipid composition A and B, respectively, are mixed together at a Y junction (Figure 4A(i)) immediately followed by the vesicle formation junction (Figure 4A(ii)).

To visualize lipid mixing control using our device, we made up two lipid-octanol phases each doped with fluorophore-labeled lipids (LipidA: DOPC:NBD-PE 98:2 mol % (green) and LipidB: DOPC:LissRhod-PE 98:2 mol % (red)). To operate the “on-the-fly” membrane composition control device, we tuned the flow rates of each of the four input phases (IA, LipidA, LipidB, and OA, see Figure 4A) until vesicle formation was achieved. Then, by varying the flow rates of each of the LipidA and LipidB phases, *Q*_A_ and *Q*_B_, respectively, while visually maintaining a similar total lipid-octanol flow rate, *Q*, at the vesicle production junction, we were able to vary the proportion of each of phase LipidA and LipidB, *x*_A_ and *x*_B_, respectively, delivered to the vesicle production junction. In Figure 4B(i), we display three different relative flow rate conditions: *Q*_A_≫ *Q*_B_, *Q*_A_ = *Q*_B_ and *Q*_A_ ≪ *Q*_B_. In each panel, LipidA and LipidB phases are shown in green and red, respectively.

Next, we probed the on-chip lipid mixing efficiency by comparing the labeled lipid composition (i.e., the ratio of NBD-PE:LissRhod-PE) of the lipid-octanol phase exiting the mixing junction with the composition of the produced vesicles in each case. We produced samples of vesicles for each composition by maintaining a given *Q*_A_:*Q*_B_ ratio for 10 min and pipetting the vesicle solution out from the outlet into separate custom-made observation wells. Between collection of each vesicle solution, first the LipidA:LipidB flow rate, *Q*_A_:*Q*_B_, was adjusted to the next value, and then vacuum was applied to the outlet to remove any vesicles from the previous sample. We measured the relative flow rates *q*_A_ = *Q*_A_/*Q* and *q*_B_ = *Q*_B_/*Q* by measuring the relative flow cross section of LipidA and LipidB phases during operation under a fluorescence microscope (see Figure S7). We then used the relationship *x*_*i*_ = *q*_*i*_ to determine the NBD-PE and LissRhod-PE composition of the mixed lipid-octanol phase. The samples of vesicles were imaged using a confocal microscope, and the NBD and LissRhod fluorescence (*I*_NBD_ and *I*_Rhod_, respectively) quantified in each case (see Experimental Methods). Representative images of vesicles for the three flow rate conditions investigated are displayed in Figure 4B(ii).

If lipid mixing of labeled lipids occurs effectively, then the ratio of *I*_NBD_:*I*_Rhod_ in the vesicles ought to be linearly proportional to the ratio of the flow rates *Q*_A_:*Q*_B_used to produce each vesicle samples. Therefore, we calculated *I*_NBD_/*I*_Rhod_ for each vesicle across the three flow conditions and plotted histograms (Figure 4B(iii)). Figure 4B(iv) shows a clear linear relationship between mean *I*_NBD_/*I*_Rhod_ and *Q*_A_:*Q*_B_, indicating that the composition of labeled lipids in the lipid-octanol phase dictates the resultant bilayer composition of the vesicles.

### DNA-Based Surface Charge Density Probe Reveals “on-the-Fly” Mixing Is Effective for Preparing Vesicles with Different Charge Lipid Fractions

While our results indicate that mixing of different PE lipids is effective on chip, these lipids are similar biophysically (packing and charge) and are likely to behave similarly in the lipid-octanol phase. However, vesicle membranes are regularly produced with lipids of varying biophysical properties, such as those with different headgroups. To investigate whether “on-the-fly” lipid composition control was effective at preparing vesicles with a range of charged lipid compositions, we applied our membrane surface charge density probe, 1C-DNA-Cy5, to compare vesicles produced by preparing lipid-octanol phases “off-chip” and “on-chip”.

**Figure 5:**
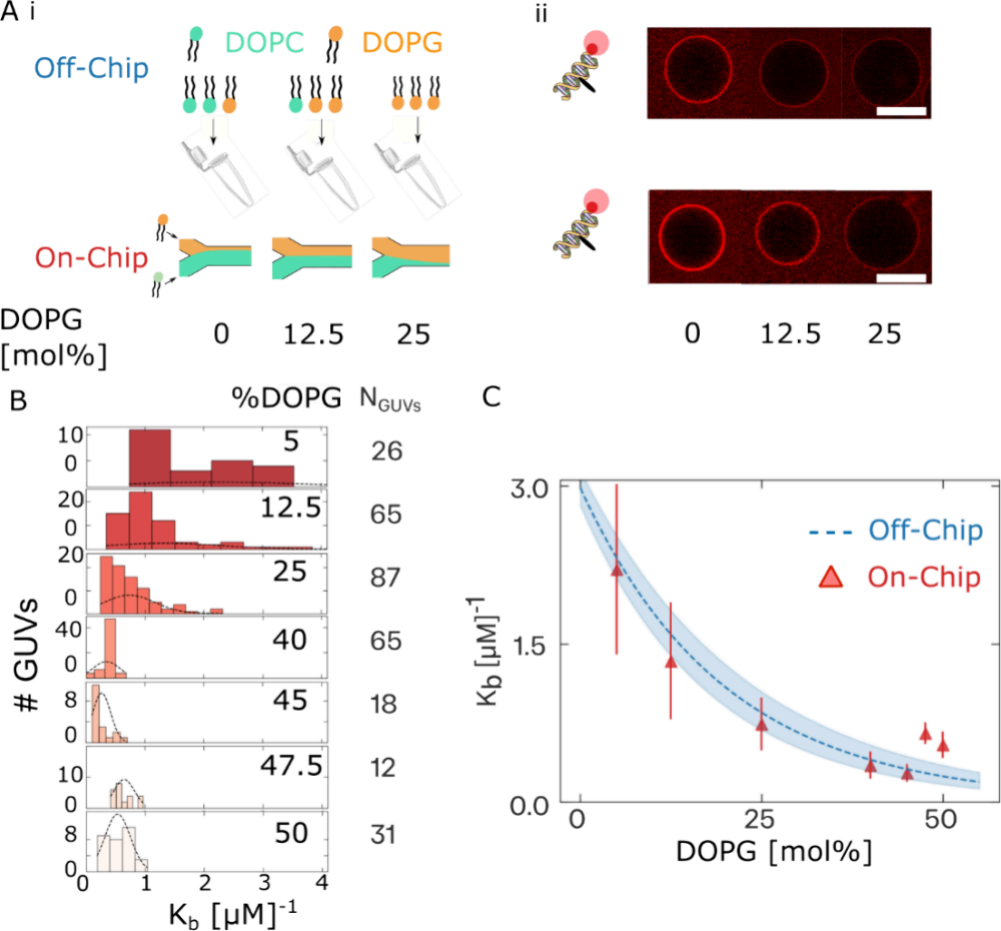
1C-DNA surface charge density sensing characterization validates “on-the-fly” vesicle membrane composition control. We characterized “on-the-fly” vesicle lipid composition control using our fluorescent probe of membrane surface charge density, 1C-DNA-Cy5. (A)(i) Illustration of the comparison of charged lipid mixing efficacy on-chip and off-chip. Vesicles of different DOPG fractions were produced either from lipid-octanol phases made up “off-chip” (top panel) or from on-the-fly mixing on-chip (bottom panel). (ii) Confocal micrographs of representative vesicles in each case after equilibration with 1C-DNA-Cy5 ([DNA]_0_ = 500 nM). Scale bar: 20 μm. (B) Distributions of 1C-DNA-Cy5/membrane binding constants, *K*_B_, for different DOPG lipid-octanol phase fractions prepared by “on-the-fly mixing”. The trend of decreasing *K*_B_ with increasing DOPG phase implies an increased membrane anionic surface charge density with increasing lipid-octanol phase DOPG fraction similar to that observed for off-chip mixing (Figure 3A). (C) Plot of mean *K*_B_ for each PG fraction prepared by on-chip mixing (red) overlaid with the fit to the mean *K*_B_ (blue, dashed) for each composition measured off-chip (see Figure 3C). Close agreement between the surface charge density of vesicles produced by on-chip mixing and off-chip methods validates the technique of “on-the-fly” lipid mixing control.

To prepare vesicles using the “off-chip” method, we made up lipid-octanol phases of different DOPG molar % and produced vesicles using the three-phase OLA method (see Experimental Methods). For each DOPG composition, we quantified the binding constant *K*_B_ with the probe 1C-DNA-Cy5 as described in the previous sections. Then, we repeated the procedure, but this time, we employed the “on-the-fly” lipid mixing device to prepare each vesicle composition, with LipidA and LipidB phases made up as DOPC:NBD-PE (99.9:0.1 mol %) and DOPC:DOPG (1:1 mol %) (see Figure 5Ai). As in the previous section, we measured the DOPG composition of the mixed lipid-octanol phase by resolving the relative flow rates of the NBD containing and fluorophore free phase (see Section S7). Extracted *K*_B_ estimates for single vesicles are displayed in Figure 5B along with their DOPG membrane fraction estimated by the flow rate ratio employed during their formation. Comparison of the trends in *K*_B_ with increasing DOPG for both methods (Figure 5C) indicates that “on-the-fly” mixing of lipid-octanol phases is equivalent to preparing lipid compositions off-chip. The principle of microfluidic mixing may also equally apply to the mixing of an increasing number of different lipid-octanol stocks, meaning that similar microfluidic devices could be used to efficiently create libraries of artificial cell lipid composition/function relationships.

## Conclusions

In summary, we designed a fluorescence probe for quantifying the surface charge density on individual vesicle membranes by employing a simple DNA-based probe. We explored the mechanism of association of our DNA probe with vesicle membranes of different surface charge densities, revealing a simple Langmuir isotherm type binding relationship. This revelation allowed us to establish the binding constant, *K*_B_, that characterizes the DNA probe association to the vesicle as a metric of membrane surface charge density. A proportionality between extracted DNA electrostatic repulsion energy and zeta potential of LUVs of the same DOPG compositions implies that our technique may prove useful in quantifying surface potentials of single GUVs. The technique for quantitatively studying surface charge density we have laid out here opens the door to single artificial cell studies of a wide variety of membrane processes, such as protein binding, charged entity transport, and membrane mechanical properties. Importantly, as our method is based on fluorescence imaging, many individual GUVs can be measured in parallel, which will be critical for efficient biophysical analysis of large vesicle composition libraries.

As our DNA sensor comprises only four single DNA strands with two chemical modifications, spanning 48 base pairs, it is orders of magnitude cheaper than similar DNA origami surface charge density probes and peptide-based probes. Indeed, we estimate the cost per vesicle sample measured as ∼£0.05, meaning that our probe is likely to be financially accessible to many research laboratories. Additionally, in comparison to similar methods based on cell-penetrating peptides, DNA-based probes with single cholesterol modifications have been found not to compromise membrane integrity to ions.^13,19^ Moreover, as the DNA itself is the surface charge density sensor and the fluorophore merely the *transducer* of the surface charge density into an optical signal, researchers may choose from a range of highly stable commercial fluorophores with a range of wavelengths, enabling the concurrent surface charge density sensing with optical probes for other artificial properties like transport. A further benefit of the DNA-based nature of our technique is that the DNA sensing module may be simply optimized or combined with other sensing modules using DNA nanotechnology without the need for familiarity with potentially technical synthesis chemistry.

Finally, we used our membrane surface charge density assay to validate the development of a new microfluidic method of vesicle formation, which introduces microfluidic mixing junctions to control the lipid composition of vesicles produced “on-the-fly”. Our demonstrations of the principle of preparing lipid compositions on a chip will enable the future generation of large-composition libraries by scaling up the number of inlet lipid channels. Moreover, in combination with our fluorescence surface charge density assay, as well as other similar biophysical assays, we can create complete pipelines for efficiently generating large information rich and multidimensional data sets. In particular, given the advent of machine learning methods, such data sets are likely to be increasingly relied upon for progress in the engineering of artificial cells.

## Experimental Methods

### Materials

1,2-Dioleoyl-*sn*-glycero-3-phosphocholine (DOPC), 1,2-dioleoyl-*sn*-glycero-3-phospho-(1-ac-glycerol) (sodium salt; DOPG), 1,2-dioleoyl-*sn*-glycero-3-phosphoethanolamine-*N*-(7-nitro-2–1,3-benzoxadiazol-4-yl) (ammonium salt; NBD-PE), and 1,2-dioleoyl-*sn*-glycero-3-phosphoethanolamine-*N*-(lissamine rhodamine B sulfonyl) (ammonium salt; 18/1 Liss Rhod PE) were purchased from Avanti Polar Lipids dissolved in chloroform to final concentrations of 25 mg/mL (DOPC and DOPG) and 2 mg/mL (NBD-PE and Liss Rhod PE). Fluorophore- and cholesterol-modified DNA strands were synthesized and purified (HPLC) by Integrated DNA Technologies (IDT) and received at 100 μM in IDT TE Buffer at pH 8. 1-Octanol was purchased from Sigma and used as received. Polydimethylsiloxane (PDMS; Sylgard 184) was purchased from Dow Corning and used as received. Phosphate-buffered saline (PBS) was purchased from Sigma as tablets and dissolved in Milli-Q water according to the manufacturer’s instructions, and pH was measured.

### Microfluidic Device Design

We used a microfluidic method to produce vesicles throughout this study, based on the octanol-assisted liposome assembly (OLA) method (Figure 1A).^23^ The description of the chip design principles has been described in several papers and is elaborated in the Supporting Information.^21−24^ Briefly, vesicles are formed by first forming water/oil/water double emulsion droplets consisting of inner aqueous (IA)/lipid octanol (LO)/outer aqueous (OA) phases. Octanol is used as the solvent for all lipid compositions and spontaneously de-wets from the middle phase of the double emulsion leaving a lipid bilayer, completing the vesicle formation (see Figure 1A). We designed an extension of this method in which microfluidic Y junctions are included in the lipid-octanol channels directly preceding the OLA formation junction allowing the mixing of two distinct lipid-octanol, LipidA and LipidB (Figure 4a). The principle of Y junction mixing is detailed in Section S8. Additionally, a video demonstration of GUV production after lipid mixing is supplied in the Supporting Information.

### Microfluidic Device Operation

Microfluidic devices were operated on a Nikon Ti2 Eclipse inverted microscope. In- and out-of-plane motion was controlled by manual micrometre stages. Applied pressures were controlled using a four-channel OB1 pressure controller (Elveflow, Elvesys, France) and were tuned as required using the ESI software (Elveflow, Elvesys, France). To operate the on-the-fly lipid mixing OLA device, four independent pressure channels were used corresponding to the IA, LipidA, LipidB, and OA flows. Fluidic reservoirs were made up into 1.5 mL Eppendorf tubes and placed in a four-port fluid manifold (Elveflow) that allowed connection of each reservoir to its own pressure port on the pressure controller. The reservoirs were connected to the microfluidic device through polymer tubing (Tygon microbore tubing 0.020 in. ID, 0.060 in. OD, Cole-Parmer, UK) and connector tips (isolated from dispensing tips, Gauge 23 blunt end, Intertronics). Camera acquisition was achieved using a Nikon DS-Fi3 CMOS camera and controlled using Nikon proprietary software, NIS-Elements, using 10× magnification.

In all experiments, the inner aqueous solution was 500 mM sucrose, PBS pH 7, and the outer aqueous 50 mg/mL Kolliphor P 188, 500 mM glucose, PBS pH 7. Lipid-octanol phases were prepared by mixing appropriate molar lipid ratios from stocks in a glass vial, evaporating the chloroform solvent using a gentle stream of nitrogen, and then resuspending in 1-octanol to a concentration of 15 mg/mL. For cases in which a single lipid-octanol phase is used to produce vesicles, the lipid-octanol solution prepared was divided equally between the LipidA and LipidB reservoirs.

### 1C-DNA Probe Binding Experiments

The following procedure was followed for each 1C-DNA/vesicle binding experiment. OLA production of the OLA from vesicles was set up as described above. For each distinct lipid-octanol phase composition prepared via the standard single lipid phase OLA method, a fresh device was prepared and used. Once vesicle production was stabilized, 30 μL of 500 mM sucrose PBS pH 7 was added to the outlet port and vesicles allowed to produce for 10 min. Meanwhile, the 1C-DNA stock was vortexed for 1 min and 40 μL of isosmotic (to the IA) buffer was prepared in a silicone incubation well (FlexWell, Sigma) on a PDMS-coated glass coverslip. Then, 40 μL of vesicles was removed carefully from the outlet using a pipet with a cut pipet tip to reduce shear forces on the vesicle. The vesicles were expelled into the 1C-DNA solution to incubate for 2 h, and evaporation was minimized by placing the incubation chamber within a sealed plastic Petri dish. To investigate the dependence of 1C-DNA probe binding as a function of ionic concentration, the experiment described above was repeated using buffers where PBS was replaced by varying concentrations of KCl solution (150, 100, and 75 mM). In each case, the inner aqueous, outer aqueous, and isosmotic glucose buffer contained equal concentrations of KCl. Note that PBS contains 137 mM NaCl, 2.7 mM KCl, 8 mM Na_2_HPO_4_, and 2 mM KH_2_PO_4._

### “On-the-Fly” Lipid Mixing Chip Operation

For “on-the-fly” lipid composition control, LipidA and LipidB relative flow rates needed to be measured and adjusted to the right ratio for the intended final lipid-octanol phase composition. To achieve this, 0.2 mol % NBD-PE was doped into LipidA or LipidB to allow visualization of the interface between the two phases at the Y junction (see Figure S7). By adjusting the pressures applied to LipidA and LipidB and observing the relative cross section of each phase (see Section S7), the final lipid-octanol phase lipid composition could be estimated. Once the desired LipidA/LipidB relative flow rate had been reached, vacuum was applied to the outlet to remove all vesicle solution from the previous lipid composition, and then 30 μL of fresh 500 mM sucrose, PBS, pH 7, was added to the outlet. Vesicle production was held stable for 10 min, and then vesicles added to the 1C-DNA probe solution as described before. Then, the next LipidA/LipidB flow rates were adjusted and the procedure repeated.

### Confocal Microscopy Imaging

Vesicle/1C-DNA binding interactions were imaged using a confocal microscope (Leica SP8 inverted confocal microscope) equipped with a white light laser. All images were taken using a 20× objective HC PL APO 20×/0.75 CS2 with a 6× zoom. NBD and Cy5 channels were imaged in sequential mode, excited using 499 and 649 nm laser lines, respectively. The optical settings were kept consistent between all calibration and binding experiments. To prevent movement of settled GUVS, the incubation wells were sealed with a coverslip, which avoided evaporation induced convective flows.

### Image Analysis

Confocal images were opened in FIJI.^40^ Vesicles were identified by eye, selecting only vesicles whose membrane maintained the same focal plane. A circle delineating the inside perimeter of the membrane was drawn by using the ImageJ circle tool. Then, using the “Make band” tool, an annulus bounding the membrane with 1 μm thickness was drawn (see Figure 2A). The mean intensity within the band was calculated and was taken as *I*_mem_. The local background intensity, *I*_bg_, was also measured (Figure 2A) and subtracted from *I*_mem_. The procedure was repeated for all vesicles identified in the images and measurements saved as .csv files. Further analysis, including distributions, plotting, and curve fitting, were achieved using custom python scripts available upon request.

## Supporting Information Available

GUV production after lipid mixing (MP4)
Microfluidic chip fabrication; illustration of octanol phase dewetting and vesicle formation; 1C-DNA design schematic; 1C-DNA probe assembly; zeta potential measurement of LUVs; calibration of intensity vs DNA concentration; calculation of maximum 1C-DNA binding by considering maximal packing; theoretical model of DNA binding to GUV membranes; determination of composition by on-the-fly mixing; microfluidic device CAD schematic; and 1C-DNA binding, which does not affect GUV shape (PDF)
