# Supplementary material for: On-the-Fly Microfluidic Control of Giant Vesicle Compositions Validated by DNA Surface Charge Sensors
Source: ACS Nano. 2025 Apr 4;19(14):13768–78. doi: 10.1021/acsnano.4c16289 (PMC12004935; doi:10.1021/acsnano.4c16289)
Supplement: Supplementary file 2 — nn4c16289_si_002.pdf [file nn4c16289_si_002.pdf]

Supplementary Information for:

On-the-fly microfluidic control of giant vesicle compositions validated by DNA surface charge sensors.

Marcus Fletcher and Yuval Elani*.

Department of Chemical Engineering, Imperial College London, Exhibition Road, London, SW7 2AZ, UK

E-mail: y.elani@imperial.ac.uk

Table of Contents

S1. Microfluidic chip fabrication..........1

S2. Illustration of octanol phase de-wetting and vesicle formation..........2

S3. 1C-DNA design schematic..........2

S4. 1C-DNA probe assembly..........3

S5. Zeta Potential measurement of LUVs..........4

S6. Calibration of Intensity vs DNA concentration..........4

S7. Calculation of maximum 1C-DNA binding by considering maximal packing.......... 5

S8. Theoretical model of DNA binding to GUV membranes 6

S9. Determination of composition by on-the-fly mixing..........10

S10. Microfluidic device CAD schematic..........11

S11. 1C-DNA binding does not affect GUV shape..........12

S1. Microfluidic chip fabrication

Master molds for the microfluidic designs were created by photolithography and soft lithography. A layer of SU-8 2025 (Microchem) was deposited using a spincoater (Laurel, UK) ((3800 rpm, 60 s with 100 rpm/s acceleration for the initial 5 s then 300rpm/s until the final speed is reached) onto a 4 in. silicon substrate (Inseto, UK). Then, the wafer was prebaked for 1 min at 65 °C, 6 min at 95 °C with temperature ramped at 5°C min. The mould was left to cool slowly on the hotplate (EMS 1000-3) for 40 minutes. Next, the wafer was placed in a UV-KUB 3 Mask aligner (Kloe, France) and an acetate mask delineating the appropriate device design placed on top, followed by a glass plate to ensure contact between mask and SU-8. The SU-8 was exposed to UV-light through the mask (7s, 365nm, 35mW/cm^{-2}). The sample was postbaked by repeating the prebaking procedure, then developed for 3 minutes in Propylene glycol monomethyl ether acetate (Sigma). Finally, the developed mould was hardbaked at 120°C for 2 minutes.

To create a negative replica from the silicon mold, we used standard soft lithography techniques. Sylgard 184 polydimethylsiloxane (PDMS) was mixed with a curing agent (9/1 w/w, Dow Corning), and the mixture was poured onto the silicon mold and baked at 75 °C for 55 min. The PDMS chip was then peeled off the mold and inlet columns created using a 0.75 mm biopsy punch (Miltex). For collection of GUVs, an outlet was cut using a scalpel to form a wide reservoir. The chip was plasma-bonded to a PDMS-coated glass coverslip using an air plasma (10 W, 25 sccm, 10 s exposure, Diener Electronic GmbH & Co. KG, Germany). The OLA formation device was further processed with a PVA coating of the post junction channel, as previously described in detail elsewhere.[1]

S2. Illustration of octanol phase de-wetting and vesicle formation.

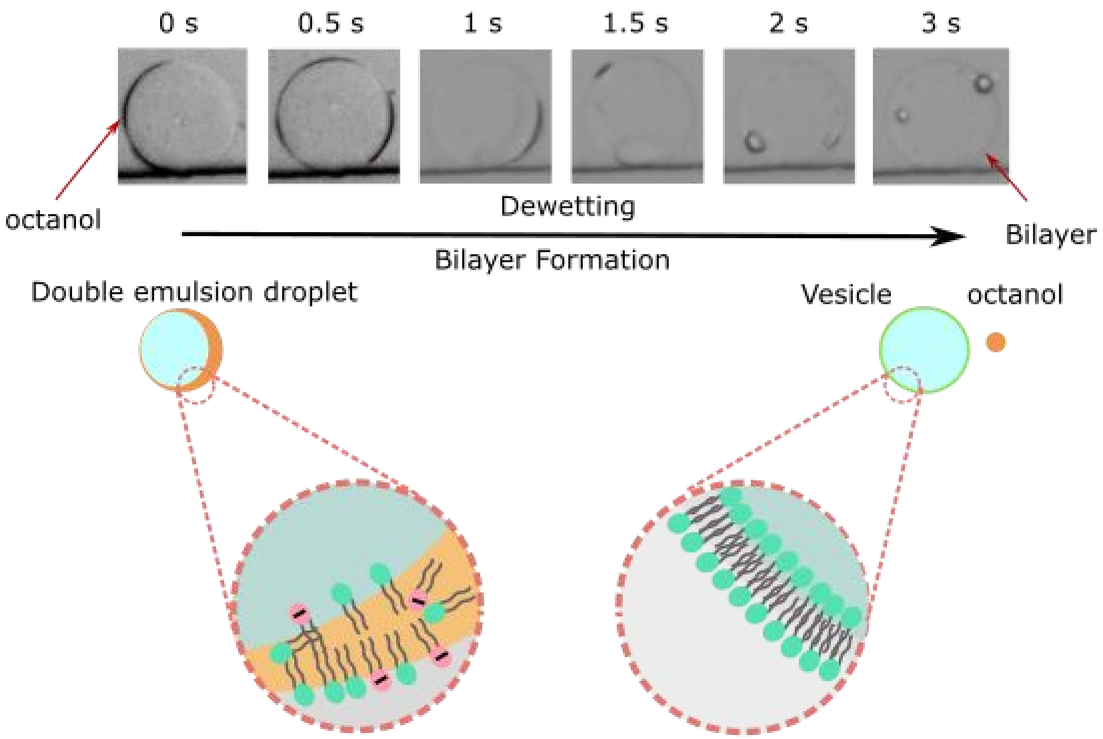

Figure S1. Annotated micrographs showing the stages of octanol de-wetting from a vesicle during Octanol-assisted Liposome Assembly. Initially Water/Lipid-Octanol/Water double emulsion droplets are formed, at which point the octanol phase (dark contrast) immediately starts to de-wet. During this process a bilayer is formed and the residual octanol phase collects into a side pocket (~ 1.5 s). Finally, the octanol phase collects into a spherical droplet allowing the bilayer to completely seal.

S3. 1C-DNA design schematic.

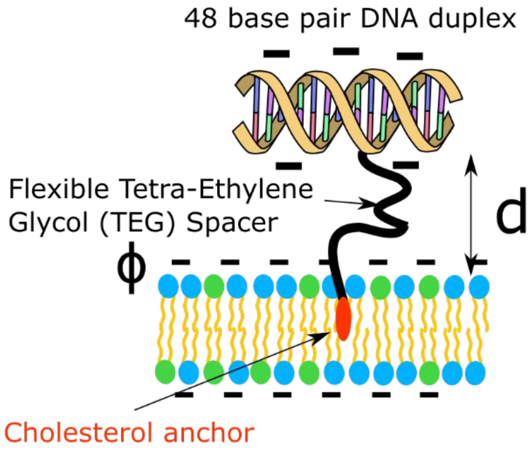

Figure S2. Schematic illustrating membrane attachment of the 1C-DNA duplex. A cholesterol anchor (red) is covalently attached to the DNA probe via a TEG linker. Due to its strong hydrophobicity, cholesterol favours insertion into the lipid membrane rather than existing free in solution. The compromise between this hydrophobic driving force for insertion, and the hydrophilicity of the DNA leads to a reversible binding process.

S4. 1C-DNA probe assembly.

The DNA probe assembly protocol was based on that performed in Morzy et al.[2] Briefly, oligonucleotides were obtained from Integrated DNA Technologies, Inc. All the strands were dissolved to a final concentration of 100 µM in IDTE buffer (10 mM Tris, 0.1 mM EDTA (Ethylenediaminetetraacetic acid), pH 8.0). Strands were then stored at -20 °C. In order to fold the designed structures, the strands were heated to 60°C for 10 minutes then immediately mixed in buffer (glucose, PBS ph7) to 2x the final concentration intended for the different experiments. In each case the glucose concentration was adjusted to balance osmotically with the sucrose in the IA (500mM glucose). The solution was vortexed for 1 minute to ensure a well mixed solution.

Four DNA strands were used to form the 1C-DNA-Dye structure, one modified with a cholesterol via a Tetra-Ethylene Glycol (TEG) linker at its 3' end, and one modified with a fluorophore (Cy5, atto532 or Atto647) at its 5' end. The sequences of each strand are given in Table S1.

Strand	Sequence (5' > 3')	Length [bp]	Modification
D1	AGTAGTATCCAT	12	
D1'	CATCGTAGCTAAAAAAGTCATACATAGATTAGAGAG	36	5'Cy5
D2	CTCTCTAATCTA	12	
D2'	TGTATGACTTTTTTAGCTACGATGATGGATACTACT	36	3'TEG Cholesterol

Table S1. DNA oligonucleotide sequences used to form 1C-DNA-Dye probe. Sequences taken from Morzy et al.[2]

S5. Zeta Potential measurement of LUVs.

LUVs were formed by hydration using the same lipid in chloroform stocks using to prepared GUVs. For each DOPC:DOPG lipid composition measured, the following protocol was used: Lipid stocks were mixed in chloroform into a glass vial to the correct molar ratio then dried with gentle nitrogen pressure. The lipid film was placed in a vacuum dessicator overnight to ensure all residual solvent was removed. Then, 1mL of 500mM glucose, PBS pH 7 was added to the lipid film and vortexed until visually no film was seen left on the glass vial walls. The final lipid concentration was 5mg/ml. The hydrated solution was then sonicated for 15 minutes at 37kHz, 100% power.

After sonication LUVs were formed using a commercially available extruder (Avanti® Polar Lipids, Avanti Mini Extruder) with the solution pressed through the extruder at least 21 times. Whatman® Filter Supports and Whatman® Nucleopore Track-Etched Membranes (100 nm) were obtained from Sigma Aldrich. The whole assembled extruded including LUVs solution was placed on a hot plate at 50 degrees for 30 minutes before performing the extrusion.

Zeta potential measurements were performed using a LiteSizer DLS instrument (Anton Paar) according to the manufacturers instructions. An Omega Cuvette specified for zeta potential measurement (Anton Paar) was washed several times with milli Q water, followed by the glucose buffer. Each LUVs sample was diluted by 10x in the same glucose buffer used to hydrate the lipid film during production. The cuvette was rinsed 1 additional time with 1mL of LUVs sample, before loading the cuvette with 1mL of sample, taking care to avoid any air bubbles. Measurements were recorded for 150 cycles at 25 °C. Model parameters are listed in Table S2.

**Model parameter**	**Value**
Conductivity (PBS)	12.8 mS/cm
Refractive index	1.332
Viscosity	0.91 mPas
Henry Factor	1.5
Relative Permittivity	76.6

Table S2. Model Parameters for Zeta Potential measurement on LUVs.

S6. Calibration of Intensity vs DNA concentration.

To measure both the concentration of membrane associated Cy5 labelled 1C-DNA, $[DNA]_{mem}$ and the free DNA in the background, $[DNA]_{ext}$, we performed calibrations of Cy5 fluorescence for both cases. To calibrate the membrane associated fluorophores we followed the method used in which a lipid labelled with the same fluorophore (Cy5) as the DNA was used as a proxy for membrane bound DNA[3]. We prepared DOPC GUVs with different fractions of Cy5 using OLA with LO phases containing different molar percentages of Cy5 labelled 18:1 PE (see main methods). For each LO phases, GUVs were settled down to the bottom of a separate custom made PDMS incubation chamber using a sucrose/glucose asymmetry (see Main Experimental) and imaged the GUVs using confocal with the same imaging settings as used in the experiments. Membrane fluorescence, I_{mem}, (see Figure S2A) was background

subtracted, and distributions of I_{mem} for each Cy5 composition plotted in Figure S2B. We observed a linear relationship between the mean GUV membrane fluorescence indicating a proportional increase in number of Cy5 fluorophores with increasing Cy5-18:1 PE inclusion.

To calibrate the background fluorescence with 1C-DNA concentration, we prepared incubation wells with different concentrations of Cy5-1C-DNA in 500mM glucose PBS pH 7 from 0 to 1 µM (see Figure S2C.)

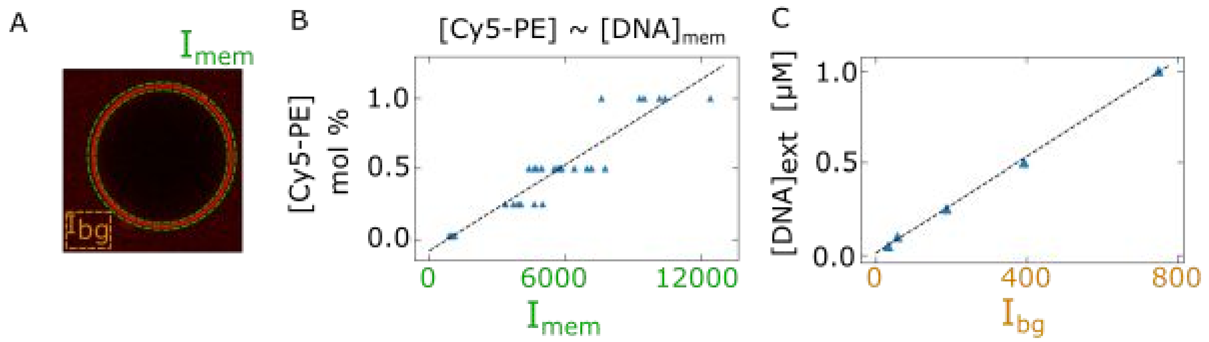

Figure S3. A Pictographic Definition of I_{mem} and I_{bg} for a representative confocal image of 1C-DNA fluorescence associated with the GUV membrane. **B** Calibration of number of membrane bound Cy5 fluorophores vs fluorescence, measured from samples of GUVs with different proportions of Cy5 labelled 18:1 PE lipid included during OLA preparation. Each blue triangle represents a single GUV. **C** Cy5-DNA fluorescence as a measure of the external free concentration of 1C-DNA (unbound) was calibrated by preparing different concentrations of Cy5-1C-DNA stock and measuring the mean intensity of images within the solution.

S7. Calculation of maximum 1C-DNA binding by considering maximal packing.

The 48 base pair DNA duplex has a length of ~ 14 nm if considered a rigid rod, which is validated when consider the DNA persistence length at 150mM ionic strength is 50 nm.[4] In addition, 1C-DNA probes bind to the membrane with the DNA axis orientated approximately parallel to the bilayer plane due to the position of the cholesterol group along the DNA duplex (see Table S1 and Figure S1).

To estimate $[DNA]_{max}$, we consider 1C-DNA probes 'maximally' packed when the centre of mass of two 1C-DNA molecules are separated by the length of the 1C-DNA duplex (i.e $\approx$ 14 nm for 48 bps). Assuming the bound DNA duplexes are free to rotate fully in the plane of the GUV, then at maximal density, each 1C-DNA molecule occupies a circular region of radius equal to 7nm. We may then estimate the maximal 1C-DNA probe density, $\sigma_{max} = 0.0065\ nm^{-2}$. Given an Area per lipid for DOPC of 67.4 $Å^2$,[5] the maximal DNA per lipid fraction, $DNA_{max} = 0.01$ i.e 1 mol%. In Figure 2b we extracted DNA_{max} for DOPC GUVs: ${DNA_{max}}^{DOPC} = 0.42 + - 0.7\ mol\ \%$. The agreement of the simple maximally packed 1C-DNA probe molecules with the observed maximal packing suggests that that 1C-DNA molecules do not influence each others binding significantly through allosteric means, further validating our simple Langmuir isotherm model of 1C-DNA probe binding (see main text).

S8. Theoretical model of DNA binding to GUV membranes.

S8.1 Relation of apparent binding constant, $K_B(n_{PG})$ to the additional electrostatic repulsion energy $\Delta\varepsilon_{elec}(n_{PG})$.

The intrinsic binding constant of 1C-DNAto the membrane, $K_{B,int}$, is related to the standard free energy change for the binding process, ΔG_0 by:

$$K_{B,int} = \exp\left(\frac{-\Delta G_0}{RT}\right) \quad \text{(S1).}$$

$K_{B,int}$ can be further related to the equilibrium concentrations of DNA bound to the membrane, $[DNA]_{mem}$ and the unbound DNA adjacent to the membrane, $[DNA]_{adj}$, according to:

$$[DNA]_{mem} = \frac{[DNA]_{max} K_{B,int} [DNA]_{adj}}{1 + K_{B,int}[DNA]_{adj}} \quad \text{(S2),}$$

where we have applied the Langmuir isotherm model validated in Figure 2 in the main text.

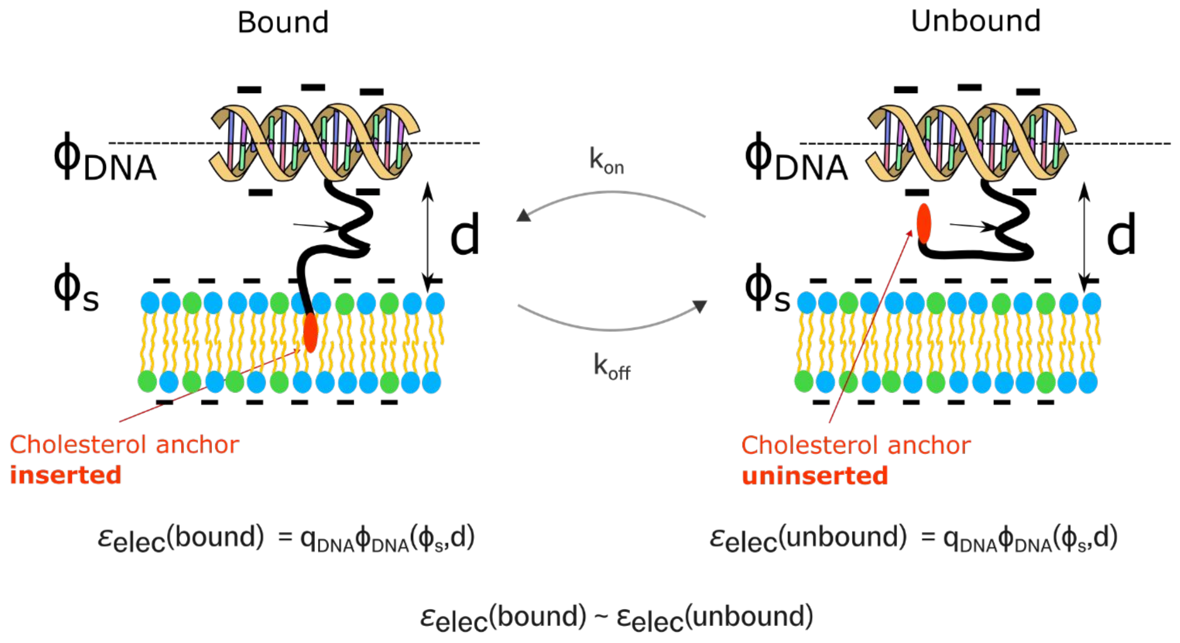

Figure S4. Molecular picture of the binding equilibrium of 1C-DNA. This binding process can be considered a dynamic equilibrium between the transition of 1C-DNA between two states i) adjacent to the membrane but without the cholesterol modification inserted into the membrane (**unbound**), and ii) with the cholesterol modification inserted into the membrane (**bound**). The equilibrium constant for this

process is defined as $K_{B,int}$, and its associated free energy change, ΔG_0, (see equation S1). As both bound and unbound states occupy approximately the same distance from the membrane, d, the electrostatic contribution to the free energy of each state, ε_{elec}, is approximately equal. Consequently, the intrinsic free energy change upon transition between these two states is independent of ε_{elec}, making $K_{B,int}$ independent of electrostatic contributions. The apparent binding constant, defined in equation 3 in the main text, and S3 here, is dependent on the electrostatic repulsion between the membrane and 1C-DNA probe due to the Boltzmann distribution of 1C-DNA at its position adjacent to the membrane.

In our experiments, we measure the apparent binding constant, K_B, which is related to $K_{B,int}$ by equation (3) in the main text:

$$K_B = K_{B,int}\exp\left(\frac{-\varepsilon_{elec}}{k_bT}\right), \text{ where } \varepsilon_{elec} = q_{DNA}\phi(d). \quad \text{(S3).}$$

The dependence of K_B on the electrostatic repulsion energy, ε_{elec}, arises due to the relationship between the concentration of 1C-DNA adjacent to the membrane, $[DNA]_{adj}$, and the measured bulk 1C-DNA concentration, $[DNA]_{free}$, which is obtained by the Boltzmann distribution:

$$[DNA]_{adj} = [DNA]_{free}\exp\left(-\frac{\varepsilon_{elec}}{k_bT}\right). \quad \text{(S4).}$$

Figure S4 details how the intrinsic binding constant may be considered to be independent of the membrane/DNA electrostatic interaction as both bound DNA and free DNA immediately adjacent to the membrane contribute the same electrostatic repulsion energy.

Accordingly, we may derive the expected dependence of the measured binding constant, K_B, on the change in electrostatic repulsion due to additional surface charge associated with increasing mole fractions of DOPG lipid:

$$K_B(n_{PG}) = K_B(n_{PG} = 0)exp\left(-\frac{\Delta\varepsilon_{elec}}{kT}\right), \quad \text{(S5).}$$

Where n_{PG} is the mole fraction of DOPG lipid in the lipid bilayer, and $\Delta\varepsilon_{elec}$is the corresponding change in electrostatic repulsion felt by 1C-DNA adjacent to the membrane. This relation appears as equation 6 in the main text.

S8.2 Model of electrostatic repulsion energy of 1C-DNA bound to GUV.

We modelled the dependence of the electrostatic repulsion energy on the zeta potential, ϕ, of the GUV surface. As the GUV surface appears approximately flat on the length scale of the 1C-DNA probe, (*O*(10nm)), we applied the analysis by Tsao[6] that derives an analytical expression for the electrostatic repulsion between a charged plate (GUV surface) and a linear charge (DNA) under Debye Hückel screening in solution:

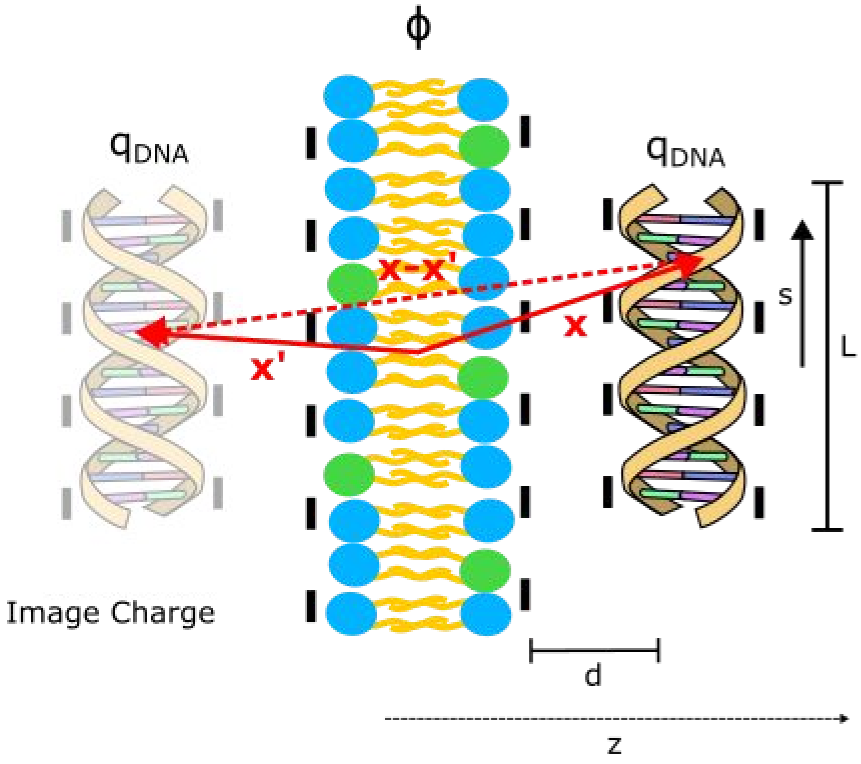

Figure S4. Model of electrostatic interaction between 1C-DNA probe (cholesterol omitted for clarity) and anionic lipid bilayer**.** As the 1C-DNA probe is designed to have a cholesterol tag along the DNA duplex we assume that it is orientated approximately parallel to the lipid bilayer on average. In this configuration the electrostatic repulsion energy, ε_{elec} , has been derived by Tsao using the method of images, resulting in equation S1.

$$\varepsilon_{elec} = \phi\int \frac{q_{DNA}}{L} e^{-\frac{z(s)}{\lambda}} ds + \frac{1}{8\pi\varepsilon_r\varepsilon_0} \iint_{-L/2}^{L/2} \frac{\frac{q_{DNA}^2}{L^2} e^{-|\boldsymbol{x}(\boldsymbol{s})-\boldsymbol{x}'(s')|}}{|\boldsymbol{x}(\boldsymbol{s})-\boldsymbol{x}'(s')|} ds ds' \quad \text{(S6),[6]}$$

where the equation variables are as defined in Figure S3. The two terms on the right hand side of this equation correspond to the interaction between the DNA and GUV surface and the image charge repulsion arising from the method of images. When the separation between the DNA duplex and the membrane is significantly larger than the debye length, λ, the second term is significantly smaller than the first term as the separation $|\boldsymbol{x}(\boldsymbol{s}) - \boldsymbol{x}'(s)| > 2\lambda$ for all points s on the duplex.

Given λ = 0.78 nm, we approximate the electrostatic repulsion energy as:

$$\varepsilon_{elec} = \phi q_{DNA} e^{-\frac{d}{\lambda}}. \quad \text{(S7)}$$

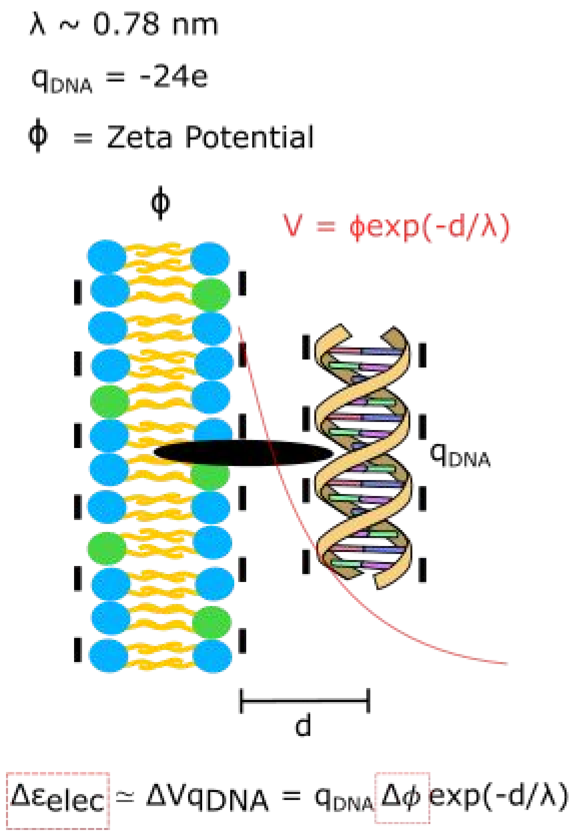

Figure S5. Model schematic for approximating the electrostatic repulsion energy between a lipid bilayer with zeta potential, ϕ, and DNA with total charge q_{DNA}. Under conditions where the distance between duplex and the slipping plane, d, does not vary significantly with $\Delta\phi$, then $\Delta\varepsilon_{elec}$ and $\Delta\phi$ are linearly related by the formula given in the bottom panel.

Given the approximately linear relationship between the change in ε_{elec}, $\Delta\varepsilon_{elec}$, and the DOPG fraction in Figure 3A (see main text), we further make the assumption in our model that the separation between d and the bilayer does not change significantly with increasing DOPG fraction. Under these assumptions, we use the measured $\Delta\varepsilon_{elec}$ to estimate the change in surface potential relative to a DOPC GUV, $\Delta\phi$, for a given DOPG molar fraction. The final model is illustrated in Figure S5. Errors in $\Delta\phi$ were estimated using the following formula, $\Delta(\Delta\phi) = \frac{\Delta d \Delta\phi}{\lambda}$,

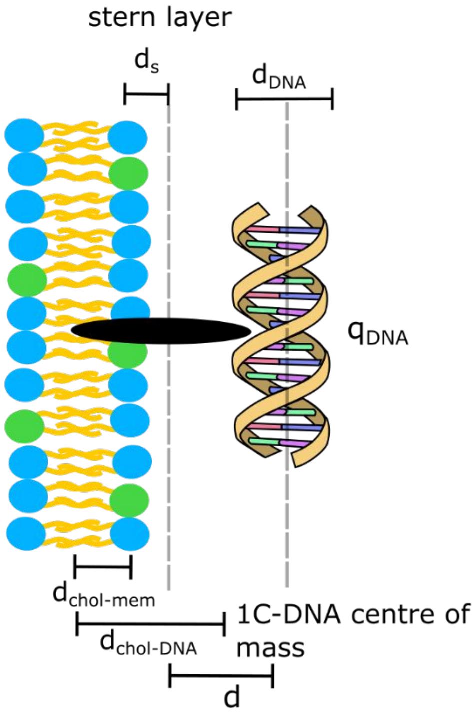

Figure S6. Estimation of 1C-DNA distance from membrane surface, d.

We estimated the distance from the centre of mass of 1C-DNA to the surface of the bilayer,d, using simulation data for a similar DNA probe studied in reference [7], where the following distances were estimated from Figure 5: $d_{chol-mem} = 0.5 \ \pm 0.03$ nm, $d_{chol-DNA} = 2 \ \pm 0.2$ nm (where errors are quoted as standard error in the mean). $d_s \sim 0.5 \ nm$ for 150mM ionic strength, estimated from the trend in Table 1 in reference [8].Finally the cylindrical radius of DNA was taken to be $d_{DNA} = 1$ nm. Taking these values, we can estimate the value d (defined pictorially above) as $d = 2 \ \pm 0.3$ nm, where the error was calculated using gaussian quadrature.

S9. Determination of composition by on-the-fly mixing.

During the operation of the microfluidic lipid mixing experiments, we measured the lipid composition by considering the relative flow rates of each of the LOa and LOb flow streams, Qa and Qb respectively (see Figure S5A). As the flow velocities are approximately the same for each stream, the relative flow rates may be approximated by the proportion of the channel cross section occupied by each stream (as illustrated in Figure S5Bi). Moreover, given the rectangular cross section of the microfluidic channels, the relative flow rate estimation may be further approximated by the proportion of channel width, d_T occupied by each phase LOa and LOb. Figure S5Bi illustrates how we measured the proportional width $\frac{d_B}{d_T}$ of the larger stream optically by tagging one lipid phase using the fluorescently labelled lipid NBD- 18:1 PE (see Main Methods).

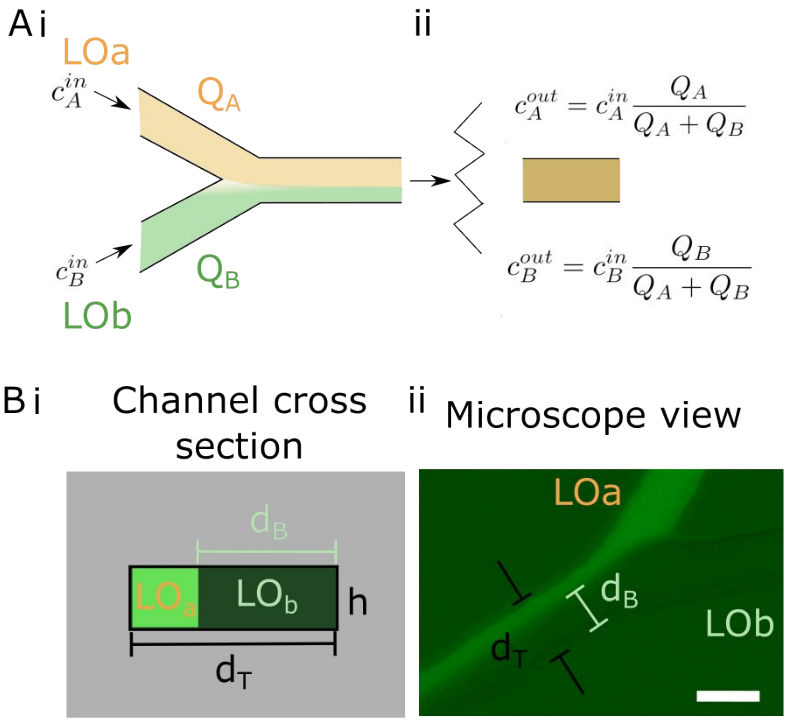

$$\frac{Q_B}{Q_A + Q_B} \approx \frac{d_B}{d_T}$$

Figure S7. Estimation of mixed LO phase composition for 'on-the-fly' microfluidic mixing. **Ai** Illustration of microfluidic Y junction used to mix phases LOa and LOb with lipid compositions c_A^{in} and c_B^{in} respectively. **ii** The final compositions of the mixed solution is given by c_A^{out} and c_B^{out} and may be tuned by changing the flow rates Q_A and Q_B of the LOa and LOb phases. **Bi** View of microfluidic channel rectangular cross section at the point of LOa (bright green) and LOb (dark) phase mixing. Due to the rectangular cross section, the relative flow rates of each phase, Q_A and Q_B, are approximately proportional to the width of each phase across the channel (d_i). **Bii** Micrograph of LO phase mixing, illustrating how the relative width of each fluid phase may be estimated. By measuring both the width of one phase e.g. d_B and the width of the combined flow, d_T, one may estimate the proportional flow rate using the relationship given (bottom panel), and thus measure both c_A^{out} and c_B^{out}. Scale bar = 30 µm.

S10. Microfluidic device CAD schematic.

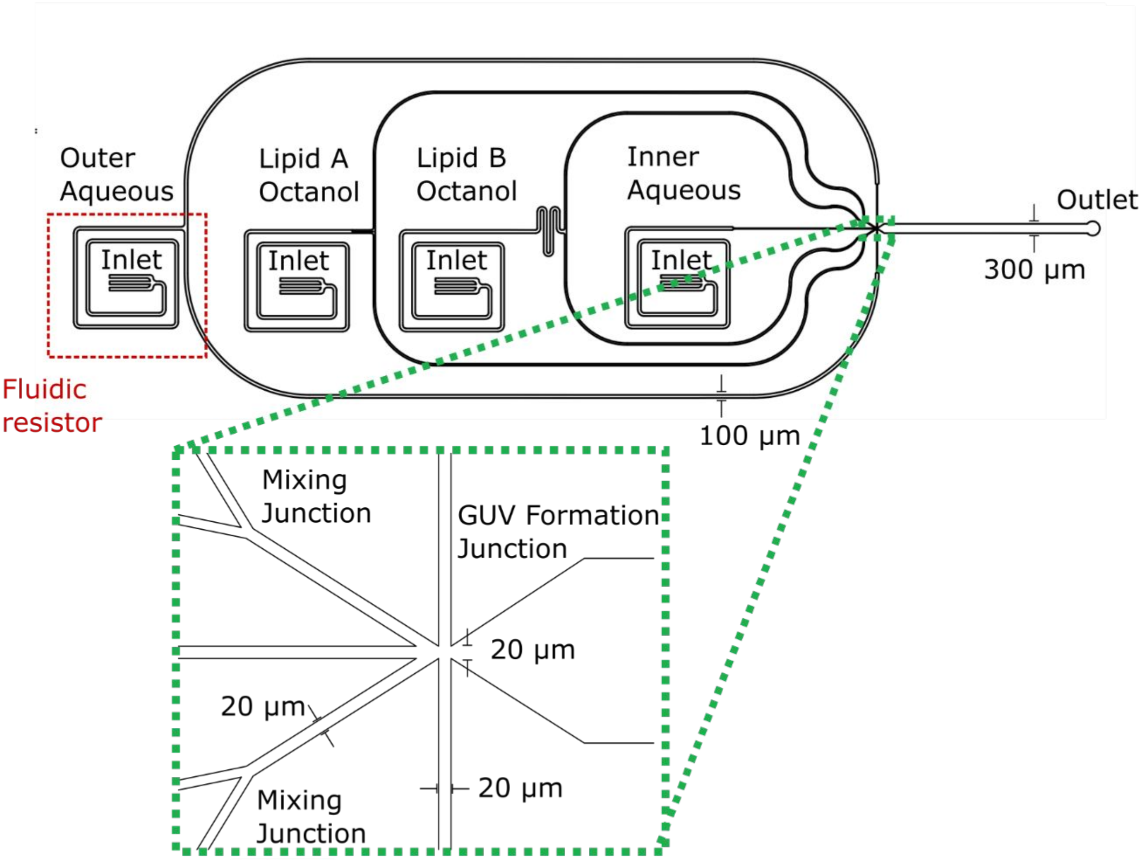

Figure S8. CAD schematic of the microfluidic device for on-the-fly lipid mixing control. Four fluidic inlets, for each fluidic phase, are shown with fork structures to aid alignment during biopsy-punching (see section S1). At each inlet, spiraling channels act as fluidic resistors to prevent back flow at the mixing junctions. The dimensions of the GUV formation and mixing junctions are shown inset.

S11. 1C-DNA binding does not affect GUV shape

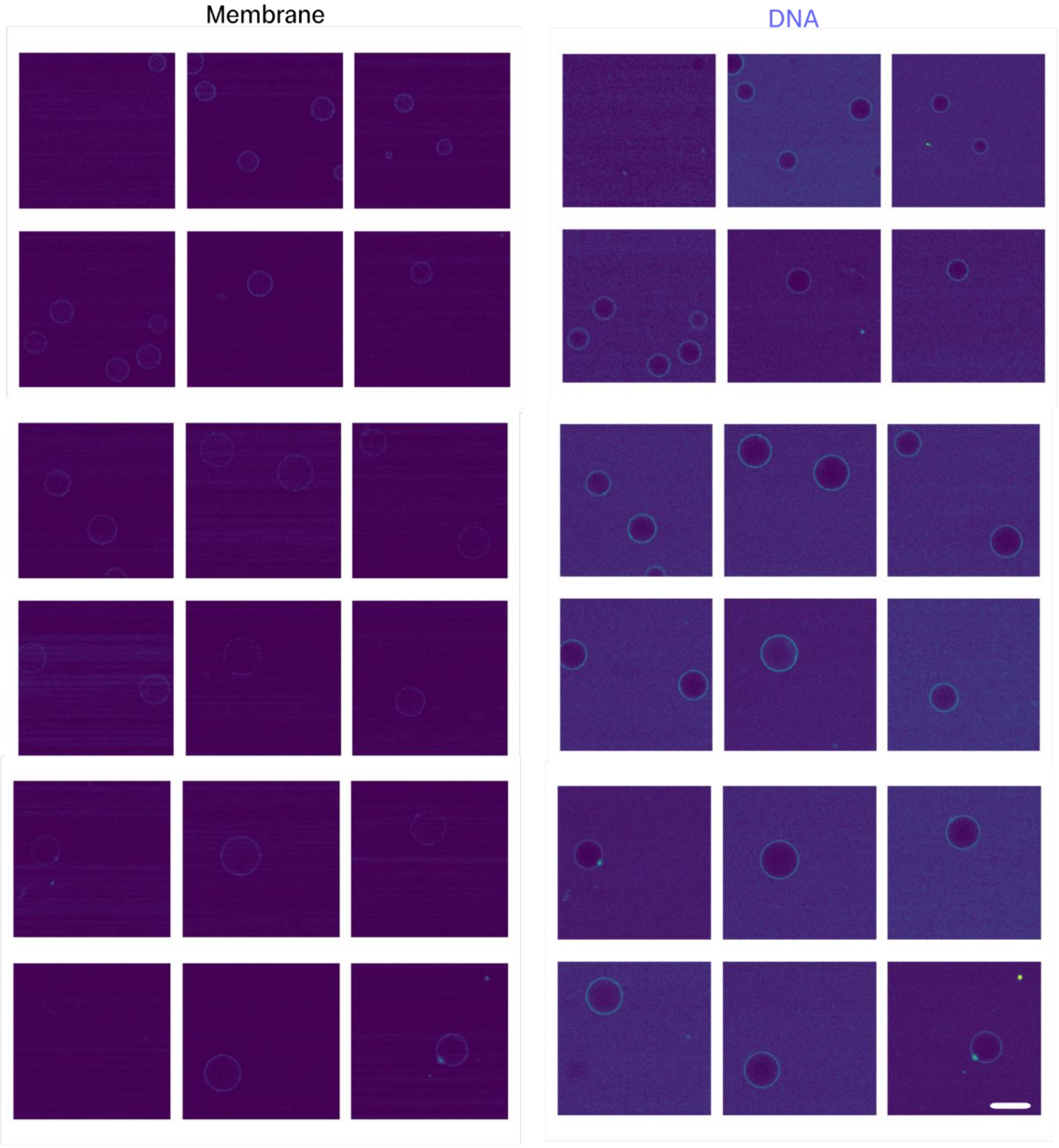

Figure S9. 1C-DNA binding does not significantly impact membrane equilibrium shape. Example images of GUVs after incubation with 1C-DNA choosing randomly from different conditions across all experimental datasets. GUVs retain their spherical shape after addition of 1C-DNA implying 1C-DNA binding (at $[DNA]_{added}$= 500nM) does not reach concentrations at which interactions may deform the membrane. Additionally, no phase separation in the membrane is evident implying cholesterol concentrations in the membrane as a result of 1C-DNA binding are too low to cause phase separation. Finally, a small proportion (~5-10 %) of GUVs imaged show bright regions in the membrane suggesting octanol side pockets. Scale bar = 30 μm.

Supplementary Video 1: A demonstration of the mixing of lipid phases on the microfluidic device

References

1. Deshpande, S., et al., *Octanol-assisted liposome assembly on chip.* Nature Communications 2016 7:1, 2016-01-22. **7**(1), 10447.
2. Morzy, D., et al., *Cations Regulate Membrane Attachment and Functionality of DNA Nanostructures.* Journal of the American Chemical Society, (2021), **143**(19), 7358-7367.
3. Steinkühler, J., et al., *Controlled division of cell-sized vesicles by low densities of membrane-bound proteins.* Nature Communications (2020), **11**(1), *11*(1), 905.
4. Mitchell, J.S., et al., *Sequence-Dependent Persistence Lengths of DNA.* Journal of Chemical Theory and Computation, (2017) **13**(4), 1539-1555.
5. Kučerka, N., et al., *Lipid Bilayer Structure Determined by the Simultaneous Analysis of Neutron and X-Ray Scattering Data.* Biophysical Journal, (2008), **95**(5), 2356-2367 .
6. Tsao, H.K., *Electrostatic Interactions of a String-Like Particle with a Charged Plate.* Journal of Colloid and Interface Science, (1998), *202*(2), 527-540.
7. Arnott, P.M., et al., *Dynamic Interactions between Lipid-Tethered DNA and Phospholipid Membranes.* Langmuir, (2018), *34*(49), 15084-15092.
8. Brown, M.A., A. Goel, and Z. Abbas, *Effect of Electrolyte Concentration on the Stern Layer Thickness at a Charged Interface.* Angewandte Chemie, (2016), *128*(11), 3854-3858
